# The Module WmABF1‐1‐WmMYB111 Manipulates the Leaf Senescence of *Welwitschia* by Integrative Regulation of Abscisic Acid Biosynthesis, Chlorophyll Degradation and Nitrogen Transportation

**DOI:** 10.1111/pbi.70290

**Published:** 2025-08-13

**Authors:** Han Xu, Qingqing Song, Junnan Wan, Jiapeng Han, Qingfeng Wang, Tao Wan

**Affiliations:** ^1^ State Key Laboratory of Plant Diversity and Specialty Crops, Wuhan Botanical Garden Chinese Academy of Sciences Wuhan China; ^2^ Sino‐Africa Joint Research Center Chinese Academy of Sciences Wuhan China

**Keywords:** abscisic acid, chlorophyll degradation, leaf senescence, nitrogen transportation, transcriptional regulation, *Welwitschia*, WmABF1‐1, WmMYB111

## Abstract

*Welwitschia mirabilis* has been well noted for its two continuously grown leaves which could survive for thousands of years in the desert. Compared to great efforts on elucidation of *Welwitschia*'s leaf adaptivity to external stresses, the regulatory mechanism of its internal leaf aging was largely unknown. Here, we revealed the module WmABF1‐1‐WmMYB111 has manipulated the proactive leaf senescence of *Welwitschia* by integrative regulation of abscisic acid (ABA) biosynthesis, chlorophyll degradation and nitrogen (N) transportation. We identified WmMYB111 as an ABA‐inducible transcription factor which could directly bind to and activate the promoters of *WmNCED6* and *WmNCED9*, creating a feedback loop to enhance ABA biosynthesis. Meanwhile, WmMYB111 positively modulated the *senescence‐associated genes* (i.e., *WmSEN1*, *WmSEN2*), *chlorophyll catabolism genes* (i.e., *WmPAO1‐1*, *WmPAO2‐1*) and *N transporter genes* (i.e., *WmNRT1.7a*, *WmNRT2.5*). Moreover, WmABF1‐1, a core ABA‐responsive element binding factor, has directly bound to and interacted with WmMYB111, shaping a transcriptional cascade as WmABF1‐1‐WmMYB111‐*WmNCEDs*/*WmSENs*/*WmPAOs*/*WmNRTs*. The WmABF1‐1 independently activated the above downstream target genes. Our findings underscored the sole module governing multidimensional regulatory mechanisms during leaf senescence, which bridged the ABA accumulation to N utilisation efficiency. To our knowledge, this is the first time transcriptional regulation was detailed in *Welwitschia* which will provide insightful clues on understanding its complex survival strategies in multi‐stressed conditions.

## 1 Introduction

**FIGURE 1:**
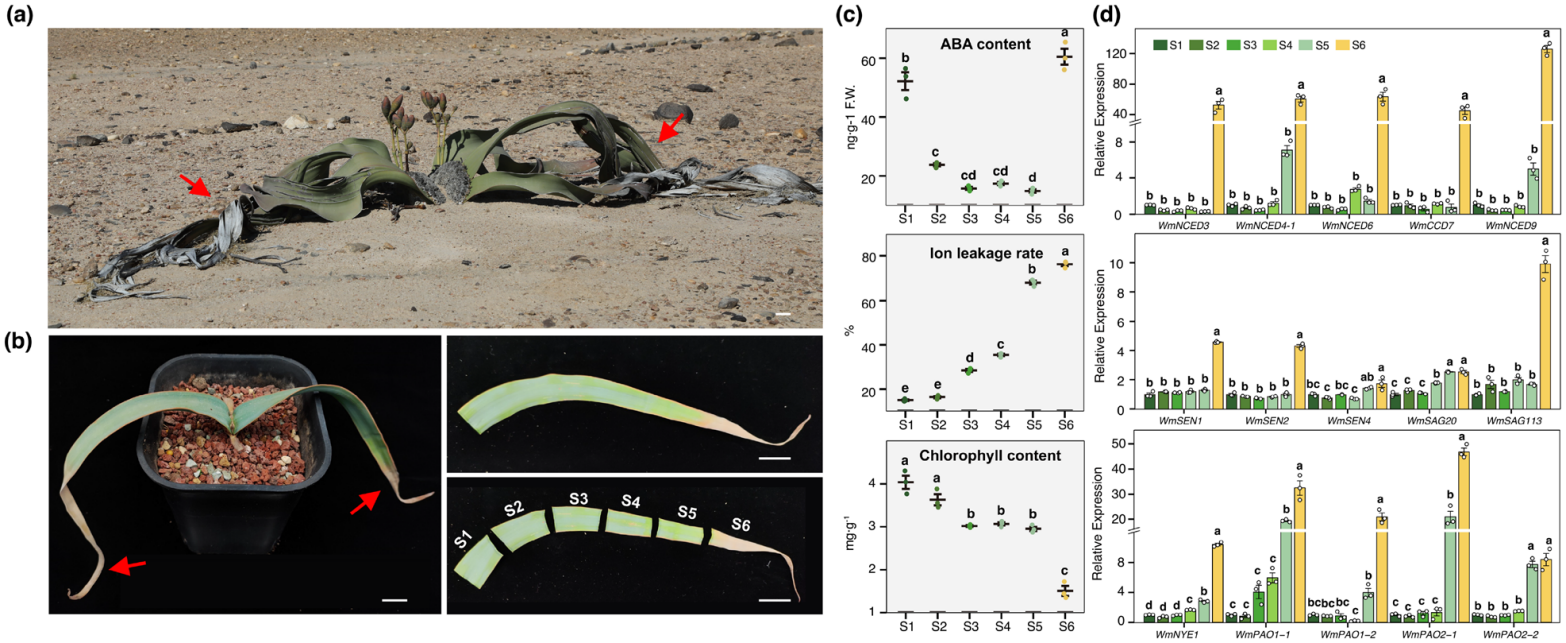
The age‐dependent leaf senescence in *Welwitschia*. (a) A female *Welwitschia* in Namib desert. Scale bar = 1 m. (b) A 3‐years old ex‐situ *Welwitschia* and the internal leaf sections ordered from S1 to S6. Scale bar = 2 cm. The observed leaf senescence was indicated by red arrows. (c) The distribution pattern of physiological features in different leaf sections. ABA contents (top), ion leakage rates (middle) and chlorophyll contents (bottom). (d) The relative expression levels of *WmNCEDs*, *WmSAGs* and *WmCCGs* in S1–S6. The means ± SE of three biological replicates and different letters above the columns of each compartment indicate significant differences with *p* < 0.05 (two‐sided of one‐way ANOVA‐HSD test driven by ‘aov’ coupled with ‘TukeyHSD’ functions).

*Welwitschia mirabilis* (hereafter *Welwitschia*) is the only species of Welwitschiaceae belonging to gnetophytes, an ancient gymnospermous lineages (Hooker 1863). The species presents a highly distinctive appearance generating only two leaves throughout the plant's life which can continuously elongate for thousands of years (Bornman et al. 1972; Willert 1985). *Welwitschia* is mainly distributed in the desert that crosses the boundary between southern Angola and northern Namibia with annual precipitation of < 50 mm (Bombi et al. 2021). In Afrikaans, the plant is thus named ‘tweeblaarkanniedood’, meaning ‘two leaves that cannot die’ (Figure 1a).

On past decades, great efforts have been made to understand *Welwitschia*'s adaptivity to hostile environments and, in particular, its developmental pattern as shaping of two ever‐growing leaves (Rodin 1958a, 1958b; Bornman 1977; Schulze et al. 1980; Pham and Sinha 2003). Unlike typical plants where leaf senescence starts at the tip and spreads to the base (Gan 2018), the leaf senescence of *Welwitschia* was confined to the tips, with the rest staying green (Figure 1b, Figure S1a). The co‐expression of *KNOTTED‐like homeobox Class 1* and *ASYMMETRIC LEAVES1/ROUGHSHEATH2/PHANTASTICAs* has been considered mainly attributable to a shifted mode of leaf development, leading the leaf growth from determinate to indeterminate in *Welwitschia* (Pham and Sinha 2003; Wan et al. 2021; Tsuda et al. 2024). Genomic survey further indicated a genetic basis for the occurrence of long‐lived leaves, such as the expansion of transcription factors (TFs) (e.g., R2R3‐MYB, bHLH) and modified pathways of DNA damage (Wan et al. 2021). In contrast to our great curiosities about such amazing species, few genetic experiments have been performed to interrogate the molecular regulation underpinning *Welwitschia* leaf longevity and/or senescence.

Leaf senescence is triggered by various internal and external factors, such as programmed cell death, phytohormones and multiple stresses (Gan 2018; Woo et al. 2019). Throughout leaf aging, the phytohormones including ethylene, strigolactones, jasmonic acid, salicylic acid and abscisic acid (ABA) have been shown to have diverse impacts (Huang et al. 2022). Among these, ABA was extensively involved in plant adaptation and leaf senescence, primarily operating in two phases (i.e., biosynthesis and signalling transduction) (Chen et al. 2020). ABA is initially derived from the carotenoid pathway where β‐carotene is converted into 9′‐cis‐neoxanthin and 9′‐cis‐violaxanthin (North et al. 2007). The enzyme 9‐cis‐epoxycarotenoid dioxygenase (NCED), encoded by *NCED3*, *NCED5*, *NCED6*, *NCED9* and other *NCEDs*, acts as the rate‐limiting factor, oxidatively cleaving these compounds to ABA (Tan et al. 2003). In terms of ABA signalling, the formation of Pyrabactin resistance 1‐like/Protein phosphatases 2C (PYLs/PP2Cs) complexes allows the release of Sucrose nonfermenting 1‐related protein kinase 2s (SnRK2s) from PP2Cs' inhibition, and activating SnRK2 by autophosphorylation or other kinases (Lin et al. 2021). The activated SnRK2 would then phosphorylate ABA‐responsive element binding factors (ABFs) to facilitate ABA signalling transduction (Cutler et al. 2010). Specifically, ABF2, ABF3 and ABF4 have directly upregulated *Chlorophyll catabolism genes* (*CCGs*) and *Senescence associated genes* (*SAGs*), resulting in ABA‐induced leaf senescence (Gao et al. 2016). Besides, BrABFs (i.e., BrABF1 and BrABF4) could modulate ABA biosynthesis and chlorophyll degradation by activating *NCED3* and *CCGs* (Tan, Fan, et al. 2019). The ABA INSENSITIVEs (ABIs) were also associated with ABA‐induced leaf senescence (An et al. 2020; Yang et al. 2024).

At the transcriptional level, TFs such as WRKY (WRKYGQK domain), NAC (NAM, ATAF1/2, CUC1/2) and MYB (v‐myb avian myeloblastosis viral oncogene homologue) are broadly participating in leaf senescence (Zhang, Guo, et al. 2021; Huang et al. 2022). There are mainly four subfamilies of MYB (i.e., 1R‐MYB, R2R3‐MYB, 3R‐MYB and 4R‐MYB) in plants. In particular, the R2R3‐MYB have played various roles in regulating plant developmental processes, ranging from primary and secondary metabolism to abiotic/biotic stress responses (Stracke et al. 2001; Dubos et al. 2010; Ambawat et al. 2013). In *Arabidopsis*, MYB2, MYBL and MYBH could positively regulate leaf senescence, whereas MYB44 and MYB59 have negative impacts (Guo and Gan 2011; Zhang et al. 2011; Huang et al. 2015; Jaradat et al. 2013; He et al. 2023). In rice, OsRL3 promotes leaf senescence and slows down the plants' response to salt stress through ABA signalling pathways (Park et al. 2018). Instead, the OsMYB102 negatively regulates ABA accumulation and signalling by suppressing ABA‐responsive genes (e.g., *OsABF4*, *OsNAP* and *OsCYP707A6*) (Piao et al. 2019). Although many senescence‐associated MYBs (Sen‐MYB) have been identified, it remains largely unexplored regarding the interactions between MYB and relevant proteins during leaf senescence (Cao et al. 2022). Furthermore, senescence can facilitate the movement of nutrients from aging leaves to developing tissues for subsequent plant fitness (Avila‐Ospina et al. 2014). Chloroplast disintegration is initiated with significantly elevated co‐expression of *CCGs* (i.e., *NONYELLOWINGs* [*NYEs*], *PHEOPHYTINASEs* [*PPHs*] and *PHEOPHORBIDE A OXYGENASEs* [*PAOs*]) (Guo et al. 2021). Chloroplast proteolysis releases nitrogenous compounds like amino acids and peptides which are further transported to younger leaves, flowers, or seeds via phloem, a process known as N remobilization (Masclaux‐Daubresse et al. 2010; Dechorgnat et al. 2011). The *N transporter genes* such as *NRT1.1*, *NRT1.7*, *NRT2.5*, *NRT3.1* and *NITRATE REDUCTASE 1* (*NIA1*) are considered essential to N provision and optimising N uptake (Havé et al. 2017; Tegeder and Masclaux‐Daubresse 2018; Zhang et al. 2024). Meanwhile, the timing of leaf senescence and N remobilization is vital for N use efficiency (NUE) as either premature or delayed senescence could lead to reduced yields (Xu et al. 2012; Havé et al. 2017). However, case studies are very limited with regard to modulated leaf senescence involving N remobilization.

Here, we verified WmMYB111, a R2R3‐MYB TF, has governed the leaf senescence of *Welwitschia* by integrative regulation of ABA biosynthesis, chlorophyll degradation and N transportation. Either silencing or overexpressing *WmMYB111* could correspondingly delay or accelerate leaf senescence, which coupled with regulation of *WmNCEDs*, *WmSENs*, *WmPAOs* and *WmNRTs*. Moreover, we revealed that WmMYB111 was activated by and interacted with WmABF1‐1 (a key activator in ABA signalling pathway) to co‐regulate the above target genes, shaping a transcriptional cascade as WmABF1‐1‐WmMYB111‐*WmNCEDs*/*WmSENs*/*WmPAOs*/*WmNRTs*. Our findings have provided insightful clues on the regulatory mechanism incorporating ABA accumulation with NUE enhancement and highlighted the multi‐faceted survival strategy of *Welwitschia* in the Namib desert, where nutrients and water were extremely limited (Abrams et al. 1997).

## 2 Results

### 2.1 Characterisation of the Age‐Dependent Leaf Senescence of *Welwitschia*

*Welwitschia* was well known for its adaptivity to the hyper‐arid desert (Figure 1a) (Schulze et al. 1980; Bombi et al. 2021). According to the average growth rate of leaves (ca. 10–15 cm per year) (Willert 1985; Wan et al. 2021), we roughly estimated the ages of ex‐situ *Welwitschia* (Figure S1a). Under more tender growth conditions, we observed leaf senescence successively occurred in different aged individuals, which is compatible with observations in natural populations (Figure S1b). In general, the *Welwitschia* leaves began yellow at approximately 3 years old after germination (Figure S1a). To characterise the leaf aging, mainly six developmental stages were defined as S1–S5 for young to old (green/yellow‐green) and S6 (yellow) for senescence to death (Figure 1b). It should be noted that the age estimation was inevitably subjective to different growth conditions, but would not become an issue for the classified internal leaf sections. Also, the technically precise ages are relatively dispensable given the extremely long lifespan of *Welwitschia*.

We measured the absolute ABA contents of the six internal leaf sections and observed a gradual decline of ABA from S1 to S5, while a sharp elevation in S6 (Figure 1c). Both ABA contents and distribution patterns were consistent with our previous findings in ex‐situ individuals of older ages (Wan et al. 2021). The electrolyte leakage gradually increased, yet chlorophyll content declined, both of which echoed the change pattern of ABA levels (Figure 1c). These physiological features jointly indicated the facet of highly programmed leaf senescence in *Welwitschia*, and progressive leaf aging, which was probably driven by endogenous ABA synthesis. We examined the expression profiles of *NCEDs*, which encode the key enzymes for ABA biosynthesis (Tan et al. 2003). In all 14 identifiable *NCEDs* (Wan et al. 2021), five genes exhibited gradually increased expression during leaf senescence (i.e., *WmNCED3*, *WmNCED4*, *WmCCD7*, *WmNCED6* and *WmNCED9*) (Figure 1d, Figure S2a). Beyond that, *WmNCED6* and *WmNCED9* showed remarkably higher expression levels than *WmNCED3*, *WmNCED4* and *WmCCD7* (Figure 1d). We thus particularly focused on WmNCED6 and WmNCED9 for subsequential analyses. Likewise, among the *WmSAGs* and *WmCCGs*, *WmSEN1*, *WmSEN2*, *WmSAG113*, *WmPAO1‐1* and *WmPAO2‐1* displayed the most significant upregulations (Figure 1d, Figure S2a). All the signatures implied a proactive regulatory mechanism of age‐dependent leaf senescence in *Welwitschia*.

It is worth mentioning that a relatively high level of ABA displayed in the newly generated leaf sections (S1) (Figure 1c). The physiological features and very low expression of aging‐induced *WmNCEDs*, *WmSAGs* and *WmCCGs* suggested the high level of ABA might have no bearing on senescence; instead, it was very likely associated with the initial growth of plant tissues as seen in other plants (Xie et al. 2020; Li et al. 2024) (e.g., cell division and elongation linked with cell wall modification).

### 2.2 WmNCED6 and WmNCED9 Positively Regulate the Leaf Senescence of *Welwitschia*

**FIGURE 2:**
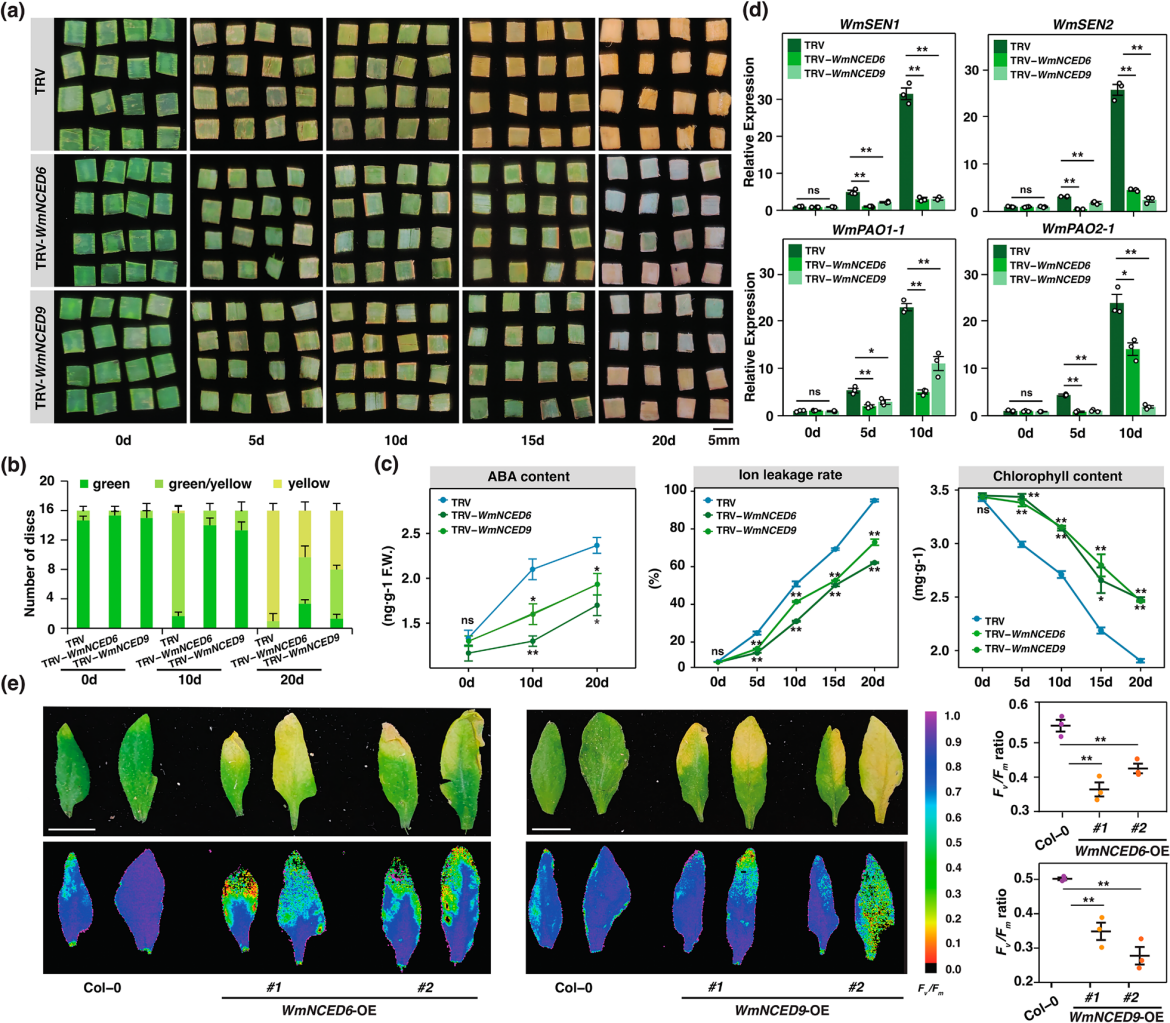
Leaf senescence of *Welwitschia* and *Arabidopsis* which were driven by either *WmNCED6* or *WmNCED9*. (a) The phenotypes of TRV‐control, TRV‐*WmNCED6*‐silenced and TRV‐*WmNCED9*‐silenced leaf discs in 0, 5, 10, 15 and 20 days. (b) Quantification of senescent phenotypes of the *Welwitschia* leaf discs. (c) Physiological indices (i.e., ABA contents, ion leakage rates and chlorophyll contents) of TRV‐control, TRV‐*WmNCED6*‐silenced and TRV‐*WmNCED9*‐silenced leaf discs from 0 to 20 days. (d) Relative expression levels of *WmSENs* and *WmPAOs* in TRV‐control, TRV‐*WmNCED6*‐silenced and TRV‐*WmNCED9*‐silenced leaf discs. For (b–d), data are means ± SE (*n* = 3 biological replicates, for each biological replicate contained at least 16 leaf discs). (e) Senescent phenotypes and *F*_v_/*F*_m_ ratios of the third and fourth rosette leaves of *Arabidopsis* wild‐type (Col‐0) and transgenic OE lines (*WmNCED6*‐OE#1,2 and *WmNCED9*‐OE#1,2). Scale bar = 5 cm. Data are means ± SE of four biological replicates, corresponding to four individuals of Col‐0 and transgenic lines shown in Figure S5. All the *p*‐values are derived from the two‐tailed Student's *t*‐test (*p < 0.05, **p < 0.01), and the ns indicates no significant difference.

The comparison of sequences and protein structure indicated that WmNCED6 and WmNCED9 were respectively homologous to AtNCED6 and AtNCED9, yet the former two were predominantly localised in chloroplasts (Figure S2b). We silenced *WmNCED6* and *WmNCED9* separately in *Welwitschia* leaf discs using virus‐induced gene silencing (VIGS). A 100 bp CDS sequence from the N‐terminus of *WmNCED6* and an 84 bp CDS sequence from the C‐terminus of *WmNCED9* were verified by BLASTN for their specificity, which were inserted into the tobacco rattle virus (TRV) vector. The RT‐qPCR confirmed that *WmNCED6* and *WmNCED9* have been effectively knocked down in TRV‐*WmNCED6*‐silenced and TRV‐*WmNCED9*‐silenced leaf discs compared to the TRV empty vector (EV) control (Figure S3a). Meanwhile, the *Welwitschia* leaves were transiently injected with the TRV‐*CLA* vector, which has been used as a phenocopy for leaf senescence‐induced de‐greening (Zhang et al. 2023). The phenotypes of TRV‐control, TRV‐*WmNCED6*‐silenced and TRV‐*WmNCED9*‐silenced leaf discs were monitored post de‐greening in the TRV‐*CLA*‐silenced areas (Figure S3b). Very slight de‐greening was observed in TRV‐*WmNCED6*‐silenced and TRV‐*WmNCED9*‐silenced leaf discs on the 15th day, while the de‐greening was much severer in TRV‐control leaf discs (Figure 2a). Moreover, the number of yellow leaf discs was considerably higher in the TRV‐control group than in the TRV‐*WmNCED6* and TRV‐*WmNCED9* silenced groups by day 20 (Figure 2b). To characterise these phenotypes, we measured ABA content, electrolyte leakage and chlorophyll levels in all transformed leaf discs. Compared to the TRV‐control, the ABA content and electrolyte leakage were lower, but the chlorophyll content was higher in both TRV‐*WmNCED6* and TRV‐*WmNCED9* (Figure 2c). The expressions of *WmSEN1*, *WmSEN2*, *WmPAO1‐1* and *WmPAO2‐1* were upregulated in TRV‐control in contrast with those of silenced *WmNCED6* or *WmNCED9* (Figure 2d). These results supported that silencing *WmNCED6* or *WmNCED9* could delay leaf senescence of *Welwitschia*.

To further investigate the functions of WmNCED6 and WmNCED9, we generated transient transformation leaf discs with overexpressed *WmNCED6* or *WmNCED9*. It showed that the de‐greening was much severer in *35S:WmNCED6* and *35S:WmNCED9* leaf discs than *35S* EV‐control (Figure S4a–c). Such phenotypes were fairly reflected by the dynamic changes in ABA content, electrolyte leakage, chlorophyll content and expression patterns of *WmSENs* and *WmPAOs* (Figure S4d–g). Accordingly, the overexpression of *WmNCED6* or *WmNCED9* could accelerate senescence in *Welwitschia* leaf discs. Beyond that, we generated transgenic *Arabidopsis* lines with overexpressed *WmNCED6* or *WmNCED9*. Two independent lines (3*5S:WmNCED6#1* and *35S:WmNCED6#2*) were introduced to assess the phenotypic traits. Compared with wild‐type (Col‐0), the *35S:WmNCED6* lines exhibited slightly earlier flowering at 15 DAG (Days after gemination) and precocious leaf senescence at 40 DAG (Figure S5a). The test of the third and fourth rosette leaves demonstrated that the photochemical efficiency of PSII (*F*_v_/*F*_m_) was lower in *35S:WmNCED6* lines than Col‐0 (Figure 2e). A significant high level of *WmNCED6* expression, ABA content and electrolyte leakage were also detected and featured as leaf senescence (Figure S5b). In addition, the expression levels of *SAGs* and *CCGs* (i.e., *AtSEN1*, *AtSAG12*, *AtPAO1* and *AtPAO2*) were dramatically increased in *35S:WmNCED6* lines (Figure S5c). Likewise, the overexpression of *WmNCED9* has promoted flowering and leaf senescence of *Arabidopsis* (Figure 2e, Figure S5d–f). Together, we claimed that both WmNCED6 and WmNCED9 could positively regulate leaf senescence either in *Welwitschia* or *Arabidopsis*.

### 2.3 Molecular Characterisation of *WmMYB111*

**FIGURE 3:**
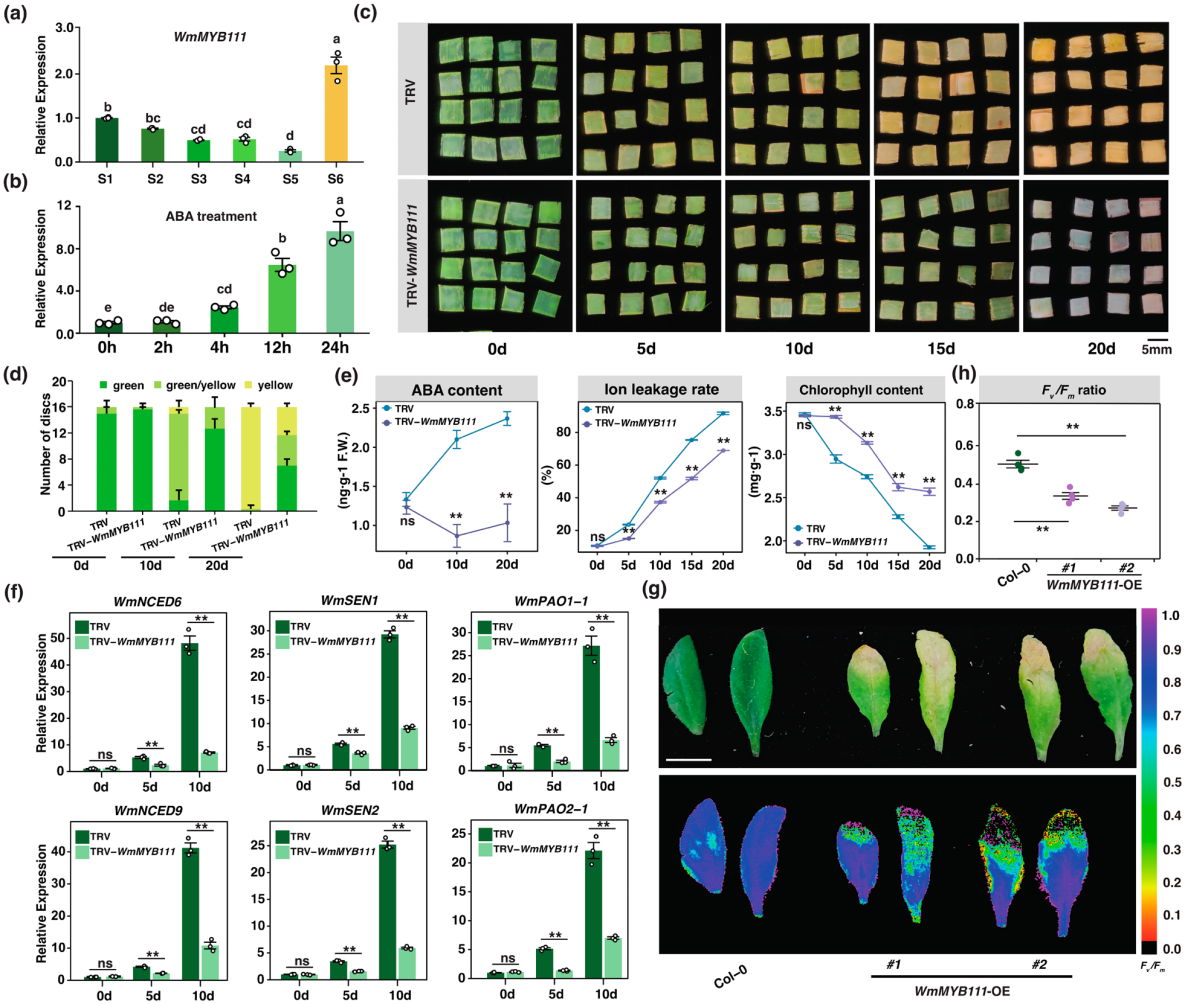
WmMYB111 modulating leaf senescence in *Welwitschia* and *Arabidopsis*. (a) The expression pattern indicated by qPCR of *WmMYB111* in S1–S6. (b) Dynamic change of *WmMYB111* expression level in different time with 50 μM ABA treatment. Data are means ± SE (*n* = 3), the significant difference at *p* < 0.05 (two‐sided of one‐way ANOVA‐HSD test conducted by ‘aov’ and ‘TukeyHSD’ functions) was shown as different letters above the columns. (c) The phenotypes of TRV‐control and TRV‐*WmMYB111*‐silenced leaf discs in 0, 5, 10, 15 and 20 days, Scale bar = 5 mm. (d) Quantification of senescent phenotypes of above leaf discs. (e) Physiological indices (i.e., ABA contents, ion leakage rates and chlorophyll contents) of TRV‐control and TRV‐*WmMYB111*‐silenced leaf discs from 0 to 20 days. (f) Relative expression levels of *WmNCEDs*, *WmSENs* and *WmPAOs* in TRV‐control and TRV‐*WmMYB111*‐silenced leaf discs. ≥ 16 leaf discs were selected as each biological replicate in (d–f), data are means ± SE (*n* = 3). (g) Senescent phenotypes and *F*_v_/*F*_m_ assays of the third and fourth rosette leaves of Col‐0 and *WmMYB111*‐OE*#1*,*2*. Scale bar = 5 cm. (h) The *F*_v_/*F*_m_ ratios of (g). Data are means ± SE (*n* = 4), as four individuals of Col‐0 and *WmMYB111*‐OE lines shown in Figure S10a. *p*‐values are analysed by using two‐tailed Student's *t*‐test (**p < 0.01), with ns indicating no significant difference.

By isolating all aging‐induced *WmNCED* promoters and predicting their TF binding sites, we noticed that the MYB binding sites (MBS) were ranked among the top three in frequency in each promoter. Among these MYBs, we found the homologies of R2R3‐MYB AtMYB111 (AT5G49330) occurred in both promoters of *WmNCED6* and *WmCCD7* (Figure S6, Data S1). We predicted that the MYB111 in *Welwitschia* might be an upstream regulator of the aging‐induced *WmNCEDs*. The phylogenetic analysis suggested W.mirabilis.18177 and W.mirabilis.22275 were the most homologous to AtMYB111 (Figure S7a). The RT‐qPCR revealed that the expression level of *W.mirabilis.18177* was highest in S6 leaf sections (Figure 3a), coinciding with the expression pattern of aging‐induced *WmNCEDs*. By contrast, *W.mirabilis.22275* was down‐regulated in the leaf aging process (Figure S7b). We thus focus on *W.mirabilis.18177* and designated it as *WmMYB111*.

The protein structure comparison and motif analysis indicated WmMYB111 was a typical R2R3‐MYB protein containing two SANT domains (Dubos et al. 2010) (Figure S7c,d). Besides, the analysis of the protein sequence indicated a nuclear localization signal site in the middle of WmMYB111, which was further verified by transient co‐infiltration of WmMYB111‐GFP with a nuclear marker in *N. benthamiana* leaves (Figure S7e). To confirm whether *WmMYB111* is also induced by ABA, we treated the leaf discs with ABA. The RT‐qPCR showed that *WmMYB111* was highly expressed after ABA treatment (Figure 3b). We further constructed the *ProWmMYB111:GUS* vector by fusing the GUS reporter gene to the C‐terminus of the MYB111 promoter. GUS assays on transiently transformed *Welwitschia* leaf discs confirmed that the activity of the *WmMYB111* promoter was substantially induced by ABA (Figure S7f). All the evidence suggested that WmMYB111 was an ABA‐inducible TF.

### 2.4 WmMYB111 Promotes ABA Biosynthesis and Leaf Senescence in *Welwitschia*

To explore the function of WmMYB111, we have silenced it in *Welwitschia* leaf discs using VIGS (Figure S8a). The phenotype indicated the silence of *WmMYB111* could delay leaf senescence (Figure 3c,d), which was characterised by lower levels of ABA content and electrolyte leakage, yet higher chlorophyll content in TRV‐*WmMYB111*‐silenced leaf discs than TRV‐control (Figure 3e). Meanwhile, the expression of *WmNCEDs*, *WmSENs* and *WmPAOs* was found downregulated in TRV‐*WmMYB111*‐silenced leaf discs (Figure 3f). More than that, silencing *WmMYB111* could still delay senescence under exogenous ABA treatment (Figure S8b). Conversely, overexpressing *WmMYB111* could promote leaf senescence, along with the significant increase of above physiological indices and expression of *WmNCEDs*, *WmSENs* and *WmPAOs* (Figure S9). We hence predicted that WmMYB111 might promote leaf senescence by upregulation of these genes. To address this, we generated transgenic *Arabidopsis* lines, *35S:WmMYB111#1* and *35S:WmMYB111#2*, which displayed early flowering and precocious leaf senescence compared to Col‐0 (Figure S10a). The features of *F*_v_/*F*_m_, ABA content, electrolyte leakage and expression of *AtNCED6*, *AtNCED9*, *AtSEN1*, *AtPAO1*, *AtPAO2* and *AtSAG12* jointly supported that overexpression of *WmMYB111* could promote leaf senescence of *Arabidopsis* (Figure 3g,h, Figure S10b,c). Despite the eFP (https://bar.utoronto.ca/) showing that *AtMYB111* was mainly expressed in petals but weakly active in leaves (Figure S10d), the phenotypes of *35S:AtMYB111#1* and *35S:AtMYB111#2* lines indicated the overexpression of *AtMYB111* has considerably promoted leaf senescence, accompanied with the upregulation of *AtSAG12* (Figure S10e).

To dissect WmMYB11's regulation of *WmNCED6* and *WmNCED9*, we respectively introduced *35S:WmNCED6* and *35S:WmNCED9* into the *Arabidopsis myb111* mutant background, generating transgenic lines *35S:WmNCED6/myb111* and *35S:WmNCED9/myb111*. Phenotypic monitoring and *F*_v_/*F*_m_ assessments revealed that leaf senescence occurred more rapidly in *35S:WmNCED6/myb111* lines than in the *myb111* mutant, along with the upregulation of *WmNCED6* (Figure S11a). Correspondingly, the ABA content, electrolyte leakage and expression levels of *AtSEN1*, *AtPAO1*, *AtPAO2* and *AtSAG12* were all notably increased in *35S:WmNCED6/myb111* lines compared to the *myb111* mutant (Figure S11b,c). It suggested that WmNCED6 acted as the downstream of MYB111. Likewise, the analyses of *35S:WmNCED9/myb111* lines attested that *WmNCED9* has functioned at the downstream of MYB111 (Figure S11d–f). By integrating all the evidence, we proposed that WmMYB111 has governed ABA biosynthesis, leaf senescence and chlorophyll degradation by regulating *WmNCEDs*, *WmSENs* and *WmPAOs*.

**FIGURE 4:**
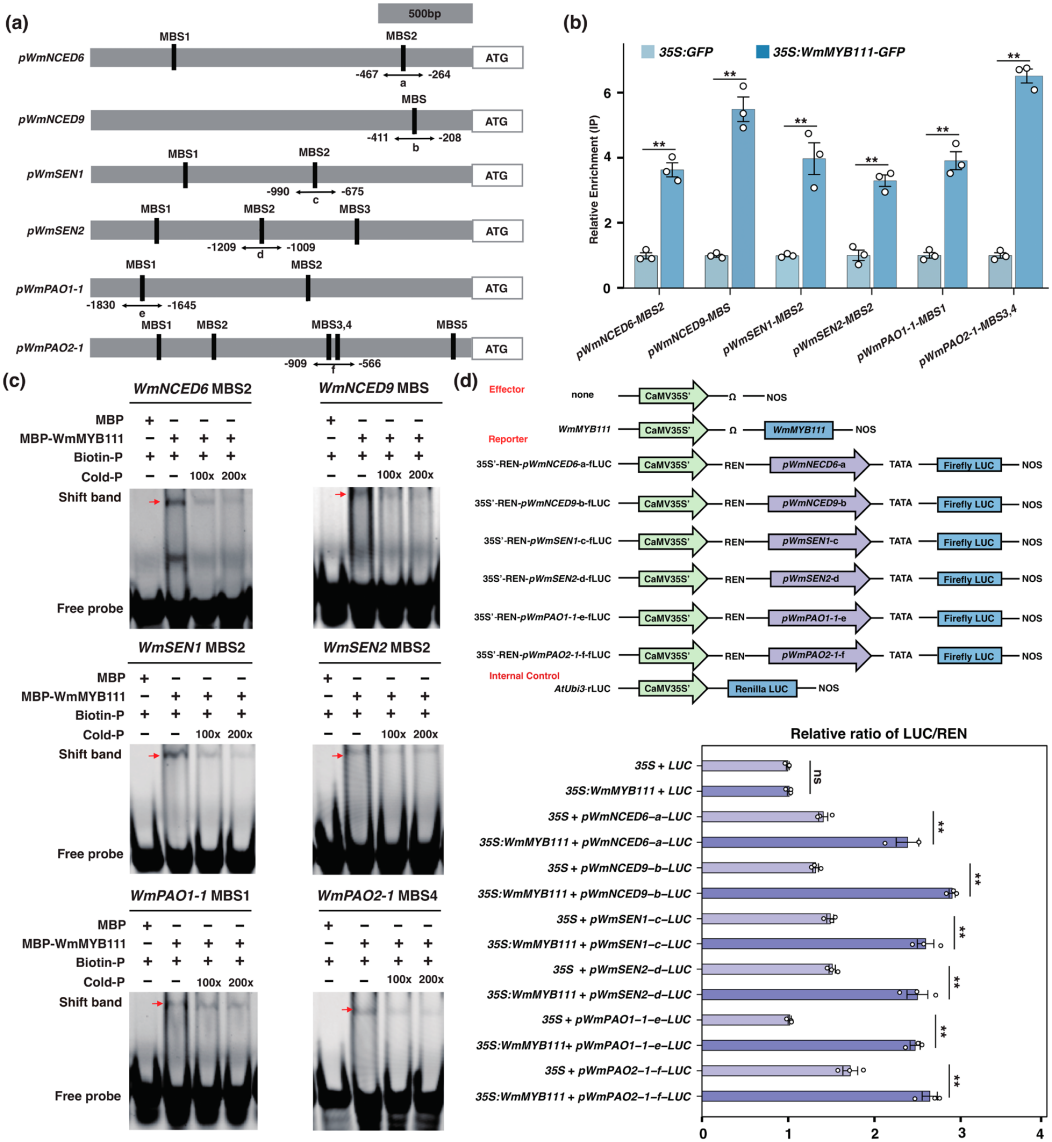
The analyses of regulatory role of WmMYB111 to its target genes. (a) The promoter regions of *WmNCED6*, *WmNCED9*, *WmSEN1*, *WmSEN2*, *WmPAO1‐1* and *WmPAO2‐1* containing MYB biding sites (MBS) clusters. The typical R2R3‐MBS (GGTAGGT) are indicated by black bar and the positions of MBS in respective promoters relative to the ATG start codon are provided. The prefix ‘*p*’ denotes the promoter and double arrows below the MBS (from ‘a’ to ‘f’) indicate the fragments used in Y1H assays (Figure S12a). (b) ChIP‐qPCR assays showing interactions between WmMYB111 and promoter fragments of above genes. (c) Interactions between WmMYB111 and promoter fragments of its target genes in vitro. The probes used in the EMSAs were designed from promoter fragments containing MBS. Purified MBP‐tagged WmMYB111 protein was incubated with 10 μM of a biotin‐labelled probe (Biotin‐P). Competition tests were performed using non‐labelled probes (Cold‐P) at 100‐ and 200‐fold excess. All shifted bands are marked with red arrows. (d) Diagram of the constructs used in the dual‐luciferase assays and the regulation of WmMYB111 on its target genes. The LUC/REN ratio represents the relative activity of the *WmMYB111* and promoters of its target genes. Data are means ± SE (*n* = 3), with *p*‐values calculated by two‐tailed Student's *t*‐test (**p < 0.01). The ns means no significant difference.

To interrogate how WmMYB111 regulates the identified downstream genes, we analysed the upstream 2 kb regions of the ATG codons and detected putative MBSs in the promoters (Figure 4a). ChIP‐qPCR analysis suggested that WmMYB111 could bind to these MBSs in vivo (Figure 4b). The EMSA (Electrophoretic Mobility Shift Assay) further confirmed that WmMYB111 could bind to these MBSs in vitro (Figure 4c). Then, using a yeast one‐hybrid (Y1H) assay, we confirmed that WmMYB111 specifically interacted with the MBS‐containing regions of the promoters of *WmNCED6*, *WmNCED9*, *WmSEN1*, *WmSEN2*, *WmPAO1‐1* and *WmPAO2‐1* (Figure S12a). We further constructed firefly *Luc* reporters of the promoter regions applied in Y1H, and co‐infiltrated them alongside effector constructs of *35S:WmMYB111* and *35S* EV‐control into the *N. benthamiana* leaves. The results demonstrated that the co‐infiltration of WmMYB111 with the promoters of these genes could significantly activate the expression of the *Luc* reporter (Figure 4d, Figure S12b).

### 2.5 WmABF1‐1 Promotes ABA Biosynthesis and Leaf Senescence in *Welwitschia*

**FIGURE 5:**
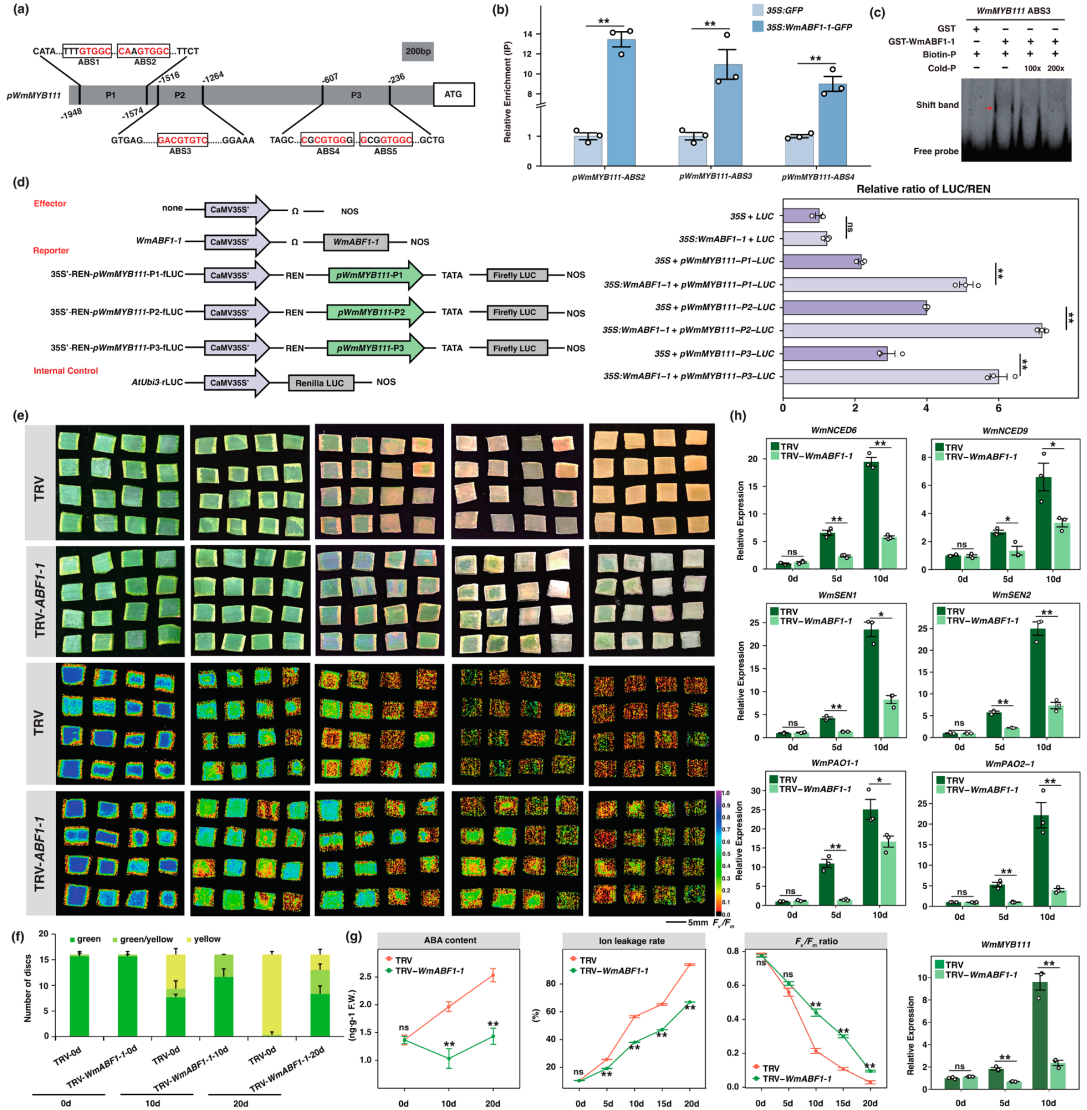
The integrative analysis of WmABF1‐1 function in *Welwitschia* leaf senescence incorporating *WmMYB111*. (a) Schematic of *WmMYB111* promoter regions containing ABF binding site (ABS) clusters. The candidate ABSs (CACGTGGC) are highlighted in red. The positions of P1–P3 fragments relative to the ATG start codon are provided. The fragments of P1 (−1948 to −1574 bp), P2 (−1516 to −1264 bp) and P3 (−607 to −236 bp) containing ABSs were used in Y1H and Dual‐luciferase assays (Figure S14). (b) ChIP‐qPCR analysis of WmABF1‐1 protein enrichment in ABS motifs of *pWmMYB111*. (c) EMSA showing the interaction between WmABF1‐1 and *WmMYB111* promoter. The probe was designed from a fragment of the promoter containing ABS3 cis‐element. GST‐WmABF1‐1 protein was incubated with 10 μM Biotin‐P. Cold‐P at 100‐ and 200‐fold were used for the competition test. The shift band is marked with arrow. (d) Diagram of the constructs used in the dual‐luciferase assays and the regulation of WmABF1‐1 on *WmMYB111*. The LUC/REN ratio represents the relative activity of the *WmABF1‐1* and *pWmMYB111* (P1–P3). (e) The senescent phenotypes and *F*_v_/*F*_m_ assays of TRV‐control and TRV‐*WmABF1‐1*‐silenced leaf discs in 0, 5, 10, 15 and 20 days, Scale bar = 5 mm. (f) The quantification of senescent phenotypes of the *Welwitschia* leaf discs. (g) Physiological features (i.e., ABA contents, ion leakage rates and *F*_v_/*F*_m_ ratios) of TRV‐control and TRV‐*WmMYB111*‐silenced leaf discs from 0 to 20 days. (h) The results of RT‐qPCR regarding the expression levels of *WmMYB111*, *WmNCEDs*, *WmSENs* and *WmPAOs* in TRV‐control and TRV‐*WmABF1‐1*‐silenced leaf discs. Above data are means ± SE (*n* = 3). The two‐tailed Student's *t*‐test were used to analyse *p*‐values (*p < 0.05, **p < 0.01), with ns means no significant difference.

The ABI and ABF have served as primary regulators of the ABA‐signalling pathway, promoting leaf senescence (Guo et al. 2021; Huang et al. 2022). In *Welwitschia*, we identified a total five copies of WmABI3, three copies of WmABF1 and one copy of WmABF2 (Figure S13a). The *WmABF1‐1* (*W.mirabilis.09338*) occurred with the highest expression level (Figure S13b) and was highly induced by ABA (Figure S13c), being suggestive of its roles in leaf senescence. The predicted protein structure of WmABF1‐1 was characterised by an additional α‐helix compared to *Arabidopsis* AtABF1, and a nuclear localization signal at its C‐terminus (Figure S13d,e). Subcellular localization of WmABF1‐1‐GFP in *N. benthamiana* verified the nuclear localization, indicating WmABF1‐1 as a TF (Figure S13f). Further, we detected five putative ABF1 binding sites (ABSs) upstream of the start codon of the *WmMYB111* promoter, which might be specifically recognised by WmABF1‐1 (Figure 5a). The ChIP‐qPCR, EMSA and Y1H assays jointly confirmed the binding of WmABF1‐1 to the P1‐P3 regions where ABSs are embedded (Figure 5b,c, Figure S14a). The dual‐luciferase reporter assays demonstrated that the luciferase activity and luminescence were significantly higher in co‐infiltrated samples of *WmABF1‐1* with P1‐P3 than in the controls (Figure 5d, Figure S14b). All these results supported that WmABF1‐1 could activate *WmMYB111* by directly binding to its promoter.

To elucidate the role of WmABF1‐1 in leaf senescence of *Welwitschia*, we performed VIGS and found that the leaf senescence in TRV‐*WmABF1‐1*‐silenced leaf discs was mild and restricted to the leaf margin by day 10, being in contrast with TRV‐control (Figure 5e, Figure S15a). The phenotypic quantification confirmed that silencing *WmABF1‐1* could delay leaf senescence (Figure 5f). Correspondingly, the ABA levels and ion leakage rates were much lower, and *F*_v_/*F*_m_ ratios were higher in TRV‐*WmABF1‐1*‐silenced leaf discs than TRV‐control (Figure 5g). Meanwhile, both *WmMYB111* and its target genes were downregulated (Figure 5h). Exogenous ABA treatment showed that silencing *WmABF1‐1* could delay the ABA‐induced leaf senescence of *Welwitschia* (Figure S15b). To the contrary, the *35S:WmABF1‐1* exhibited accelerated senescence and lower *F*_v_/*F*_m_ ratios compared to *35S*‐control when overexpressing *WmABF1‐1* in leaf discs (Figure S16a–c). The increased ABA levels and electrolyte leakage, together with the upregulated expression of *WmMYB111* and its target genes in *35S:WmABF1‐1* leaf discs, would collectively suggest WmABF1‐1 as a positive regulator of ABA biosynthesis and leaf senescence (Figure S16d–f). Meanwhile, the overexpression of *WmABF1‐1* in Col‐0 could promote flowering and leaf senescence along with elevated expression of *WmABF1‐1* and *AtSAG12* (Figure S17a,b), which is similar to *AtABF1* expression in senescent *Arabidopsis* leaves (Figure S17c). To validate the regulatory link between WmABF1‐1 and WmMYB111, *WmMYB111* was introduced into the *abf1* mutant. Compared with *abf1*, the early flowering and precocious leaf senescence of *35S:WmMYB111/abf1#1,2* indicated that WmABF1‐1 acted upstream of WmMYB111 in modulating leaf senescence (Figure S17d,e).

### 2.6 WmABF1‐1 Interacts With WmMYB111 In Vivo and In Vitro

Given that either silencing or overexpressing *WmABF1‐1* could affect the expression of WmMYB11's target genes, we anticipated a direct regulatory role of WmABF1‐1 to the downstream genes (i.e., *WmNCED6*, *WmNCED9*, *WmSEN1*, *WmSEN2*, *WmPAO1‐1* and *WmPAO2‐1*). Through analysing the promoter cis‐elements, we identified multiple putative ABSs in these genes (Figure S18a). The ChIP‐qPCR and *Luc* reporter assays showed that WmABF1‐1 could bind to and activate the promoters of *WmNCED6*, *WmSEN1* and *WmPAO1‐1*, implying co‐target genes shared by WmMYB111 and WmABF1‐1 (Figure S18b,c). It was whilst reckoned the potential interaction between WmMYB111 and WmABF1‐1.

**FIGURE 6:**
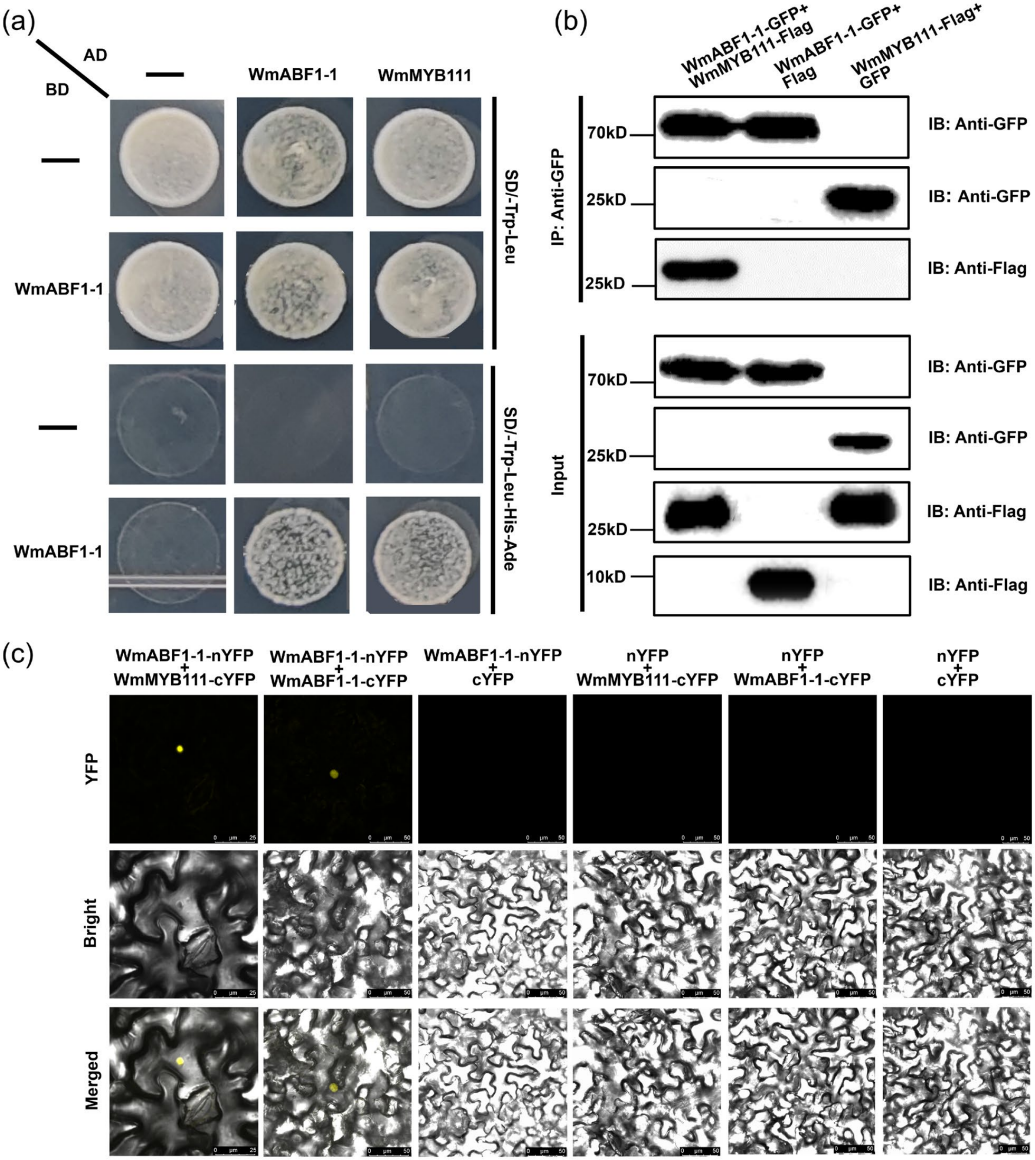
The interaction between WmABF1‐1 and WmMYB111. (a) Y2H assays show the interactions between WmABF1‐1 and WmMYB111, as well as WmABF1‐1 self‐interaction, indicated by cell growth on SD/‐Trp/‐Leu/‐His/‐Ade. Yeast grown on SD/‐Trp‐/Leu served as controls and the experiment was replicated three times. (b) Interaction between WmABF1‐1 and WmMYB111 in Co‐IP assays. Proteins were extracted from *N. benthamiana* transformed with *35S:WmABF1‐1‐GFP* and *35S:WmMYB111‐Flag* followed by immunoprecipitation with an anti‐GFP antibody. Gel blots were probed with anti‐GFP and anti‐Flag antibodies. The input served as the loading control. IB means Immunoblotting and IP means immunoprecipitation. (c) BiFC assays display nuclear interactions between WmABF1‐1 and WmMYB111, as well as WmABF1‐1 self‐interaction. Control groups included various nYFP/cYFP combinations, with no fluorescence detected. Scale bars are raged from 25 to 50 μm.

The Yeast two‐hybrid (Y2H) assays suggested that WmABF1‐1 was physically interacting with WmMYB111 as well as the self‐interaction of WmABF1‐1 (Figure 6a). We conducted co‐immunoprecipitation (Co‐IP) by transiently co‐expressing *WmABF1‐1‐GFP* and *WmMYB111‐Flag* in *N. benthamiana*. The WmABF1‐1‐GFP complexes were pulled down using GFP‐trap beads, and the interaction was examined using anti‐Flag antibodies. The results demonstrated that WmABF1‐1‐GFP was co‐immunoprecipitated with WmMYB111‐Flag, but not with the Flag‐control, suggesting the interaction between WmABF1‐1 and WmMYB111 in vivo (Figure 6b). To further explore subcellular localisation of the interaction, we constructed bimolecular fluorescence complementation (BiFC) vectors for WmABF1‐1‐nYFP, WmABF1‐1‐cYFP and WmMYB111‐cYFP. The YFP signals were detected in the nucleus but not in control groups when these vectors were transiently co‐expressed in *N. benthamiana* (Figure 6c). These results were unequivocally corroborated with the interaction of WmABF1‐1 and WmMYB111, as well as the self‐interaction of WmABF1‐1, as forming protein complex in vivo and in vitro.

### 2.7 WmABF1‐1 and WmMYB111 Promote N Transport by Directly Binding and Activating the Promoters of *WmNRTs*

**FIGURE 7:**
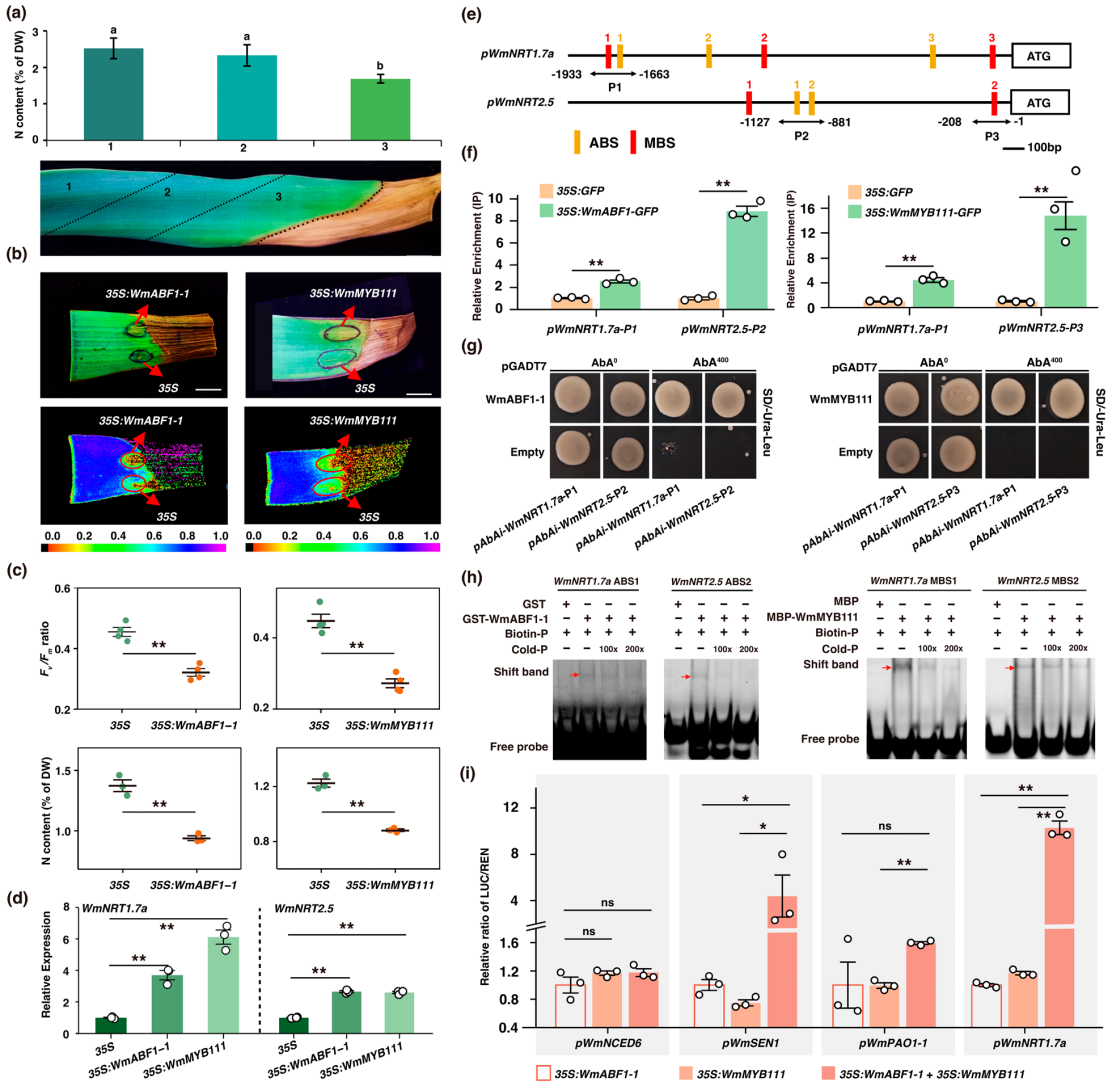
WmABF1‐1 and WmMYB111 regulating nitrogen transport by directly targeting and activating the promoters of *WmNRTs*. (a) The N contents in different *Welwitschia* leaf sections (1, 2: Green; 3: Green/yellow). Data are means ± SE (*n* = 3), with letters of ‘a’ and ‘b’ above the columns indicating the significant difference at *p* < 0.05 (one‐way ANOVA‐HSD test of two‐sided performed with ‘aov’ and ‘TukeyHSD’ functions). (b) The phenotypes and *F*_v_/*F*_m_ assays at 10th day in proximal areas of senescent sections which transiently transformed with *35S*, *35S:WmABF1‐1* and *35S:WmMYB111*. (c) The *F*_v_/*F*_m_ ratios and N contents in (b). Data of *F*_v_/*F*_m_ ratios and N contents are respectively means ± SE (*n* = 4 and *n* = 3). (d) The expression levels of *WmNRT1.7a* and *WmNRT2.5* in transformed areas. (e) Schematic of *WmNRT1.7a* and *WmNRT2.5* promoters with ABS/MBS clusters (yellow/red), indicating by double arrows (P1–P3) used for ChIP‐qPCR, Y1H and Dual‐luciferase assays (Figure S20b). (f) ChIP‐qPCR analysis of WmABF1‐1 and WmMYB111 protein enrichment in motifs of *pWmNRT1.7a* and *pWmNRT2.5*. (g) Interaction of WmABF1‐1/WmMYB111 with ABS/MBS regions of *WmNRT1.7a* and *WmNRT2.5* promoters were demonstrated via Y1H assays, based on cell growth on SD/‐Ura/‐Leu containing 0 and 400 ng·mL^−1^ AbA, respectively. (h) EMSA of interactions between WmABF1‐1/WmMYB111 and the promoters of *WmNRT1.7a*/*WmNRT2.5*. The probe was designed from a fragment of the promoter containing ABS/MBS cis‐element. GST‐tagged WmABF1‐1 and MBP‐tagged WmMYB111 proteins were incubated with 10 μM Biotin‐P. Cold‐P at 100‐ and 200‐fold were used for the competition test. All shift bands are marked with arrows. (i) Relative ratio of LUC/REN revealed the co‐expression of *WmABF1‐1* and *WmMYB111* significantly enhanced transcriptional activity of *pWmSEN1*, *pWmPAO1‐1* and *pWmNRT1.7a* compared to *WmABF1‐1*/*WmMYB111* alone. For (d, f, i), data are means ± SE (*n* = 3). *p*‐values (*p < 0.05, **p < 0.01) are analysed by two‐tailed Student's *t*‐test. The ns means no significant difference.

To ascertain whether leaf senescence in *Welwitschia* was linked with N recycling or deficiency, we measured N content in different leaf sections close to the tips. We found no obvious difference between the green sections (‘1’ and ‘2’). Interestingly, the N content dramatically dropped in green/yellow sections nearby the senescent areas (‘3’) (Figure 7a). We found *WmNRT1.7a* and *WmNRT2.5* were remarkably upregulated in ‘3’ among the identified *Welwitschia NRTs* and *NIAs* (Figure S19a). Indeed, the NRTs and NIAs have played essential roles in N assimilation and transportation (Zhang et al. 2024). Moreover, the leaf senescence of *Welwitschia* could be delayed under high nitrogen and accelerated under low nitrogen (Figure S19b).

To explore the roles of WmABF1‐1 and WmMYB111 in N transportation, we respectively injected *Agrobacterium* harbouring *35S:WmABF1‐1*, *35S:WmMYB111* and *35S* EV into leaf section ‘3’. Comparing to *35S*‐control, the leaf yellowing was accelerated in *35S:WmABF1‐1* and *35S:WmMYB111* alongside the reduced *F*_v_/*F*_m_ ratios and N contents (Figure 7b,c). The RT‐qPCR showed that *WmNRT1.7a* and *WmNRT2.5* were upregulated in the areas with injected *35S:WmABF1‐1* and *35S:WmMYB111* (Figure 7d). It thereby suggested that WmABF1‐1 and WmMYB111 may regulate N transportation during chlorophyll degradation. To confirm this, we measured N content in the leaves and seeds of *WmABF1‐1* and *WmMYB111* transgenic *Arabidopsis*, referring to a previous study (Zhang et al. 2024). The N levels in the leaves of *35S:WmMYB111#1* and *35S:WmABF1‐1#2* were significantly reduced compared to Col‐0 (Figure S20a). Besides, the *abf1* mutant showed higher leaf N content than Col‐0, and overexpression of *WmMYB111* in *abf1* decreased its leaf N content (Figure S20a). In contrast to the leaf, the N content in seeds was increased in *35S:WmMYB111#1*, *35S:WmABF1‐1#2* and *35S:WmMYB111/abf1#1* (Figure S20a). All the findings indicated a regulatory role of WmABF1‐1‐WmMYB111 in N transportation between the leaf and seed. To elucidate the mechanism of WmABF1‐1‐WmMYB111 in modulating N transportation, we analysed the promoters of *WmNRT1.7a* and *WmNRT2.5*. Multiple putative ABSs and MBSs were identified in their promoters (Figure 7e). The ChIP‐qPCR, Y1H, EMSA and *Luc* reporter assays confirmed that WmABF1‐1 and WmMYB111 could directly bind to and activate the promoters of *WmNRT1.7a* and *WmNRT2.5* (Figure 7f–h, Figure S20b). Beyond that, we co‐infiltrated *N. benthamiana* leaves with *Agrobacterium* containing *35S:WmABF1‐1*, *35S:WmMYB111* and *Luc* reporter constructs driven by target gene promoters. The LUC/REN ratios suggested that co‐expression has significantly enhanced the transcriptional activity of *pWmSEN1*, *pWmPAO1‐1* and *pWmNRT1.7a* compared to WmABF1‐1/WmMYB111 alone, suggesting a synergistic interaction between WmABF1‐1 and WmMYB111 (Figure 7i).

## 3 Discussion

As primary source organ, leaves produced necessary nutrients through photosynthetic programme. For *Welwitschia*, only two leaves persisted aboveground throughout its entire life. It would thus be of fundamental interest to understand their long‐term survival strategy on dealing with multiple‐stressed conditions. Meanwhile, the unique developmental pattern of *Welwitschia* leaf would enable the assessment of each leaf segment in a way not feasible with any other plants (Willert 1985), helping to interrogate pivotal endogenous factors (e.g., phytohormones and genes) driving the transition of leaves from young to senescence.

**FIGURE 8:**
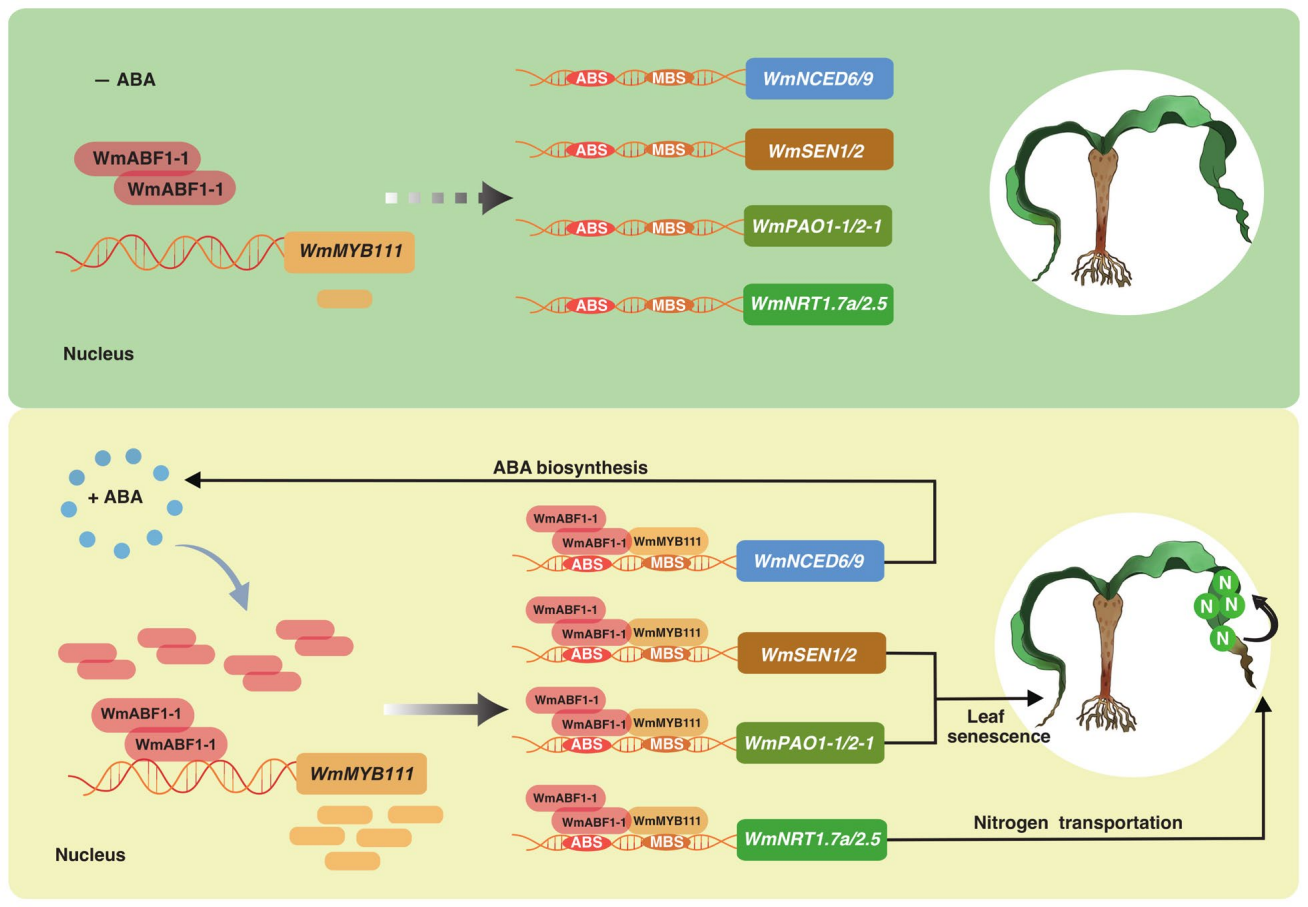
The model of the regulation of ABA‐induced leaf senescence and nitrogen transport in *Welwitschia* governed by WmABF1‐1‐WmMYB111 module. With the accumulation of ABA, the enriched WmABF1‐1 will induce the expression of *WmMYB111*. WmMYB111 would then activates ABA biosynthetic genes (*WmNCEDs*), shaping a positive feedback regulation to generate more ABA. Simultaneously, WmMYB111 activates *Senescence associated genes* (*WmSENs*) and *Chlorophyll catabolic genes* (*WmPAOs*) to trigger protein and chlorophyll degradation, leading to leaf senescence and releases of nitrogenous compounds (e.g., amino acids and peptides). Thereafter, WmMYB111 activates *N transporter genes* (*WmNRTs*) for N transportation. During this process, the self‐interaction of WmABF1‐1 and WmABF1‐1‐WmMYB111 module jointly regulate *WmNCEDs*, *WmSENs*, *WmPAOs* and *WmNRTs* to promote ABA biosynthesis, leaf senescence, chlorophyll degradation and N transportation.

Here, we inferred the cardinal regulatory role of the WmABF1‐1‐WmMYB111 module during leaf senescence (Figure 8). Although Sen‐MYBs have been reported as regulating leaf senescence and chlorophyll degradation by modulating phytohormones (Guo and Gan 2011; He et al. 2023), few of them have been recognised as regulators of ABA biosynthesis during leaf aging (Piao et al. 2019). Instead, most Sen‐MYBs were involved in the ABA perception and signalling pathway (Jaradat et al. 2013; Huang et al. 2015; Park et al. 2018). Beyond that, compared to WRKY and B3 TFs which were extensively involved in the regulation of *NCED* (Liu et al. 2015; Sato et al. 2018), the MBSs were found to overrepresent the putative TFs binding sites in all senescence‐induced *WmNCEDs* (Figure S6). We anticipated more prominent roles of MYB TFs in regulating *NCEDs* in *Welwitschia*. Indeed, the screened WmMYB111 could not only regulate ABA biosynthesis but also was responsible for leaf senescence and chlorophyll degradation (Figure 3, Figure S9). In other plants, most MYB111 homologues were mainly involved in the biosynthesis of flavonoids, phenolic acids, phenylpropanoids and abiotic stress responses (Stracke et al. 2010; Pandey et al. 2014; Li et al. 2018, 2019; Tan, Man, et al. 2019; Zhang et al. 2019; Pathak et al. 2022; Zhou et al. 2022). In *Arabidopsis*, despite the weak expression of *AtMYB111* in leaves, our results showed that both *WmMYB111*‐OE and *AtMYB111*‐OE can promote leaf senescence (Figure S10d,e). Given that both *WmNCED6*‐OE and *WmNCED9*‐OE have promoted leaf senescence in Col‐0 and *myb111* mutant (Figures S5 and S11), the regulatory module MYB111‐NCEDs was probably conserved across different seed plant lineages.

Among the core components in ABA perception and signalling pathway, the PYLs, PP2Cs, SnRK2s, ABIs and ABFs were closely related to the regulation of downstream structural genes, modulating ABA‐mediated life processes (Cutler et al. 2010). For instance, phosphorylated AtABF2 by SnRK2s could promote ABA‐induced *SAG12* expression, leading to leaf senescence in *Arabidopsis* (Zhao et al. 2016), likewise to AtABF3 and AtABF4 (Gao et al. 2016). The AtABF1, AtABF2 and AtABF4 have regulated *AtTPPI* (*Trehalose‐6‐phosphate phosphatase I*) to improve drought resistance of *Arabidopsis* through decreasing stomatal apertures (Lin et al. 2020). In addition, ABFs were responsible for chlorophyll degradation in leaves and fruit peels, activating downstream genes like *CCGs* and *ANS‐6D* (*Anthocyanidin synthase 6D*) (Gao et al. 2016; Tan, Fan, et al. 2019; Hu et al. 2019; Li et al. 2022). In *Welwitschia*, only ABI3, ABF1 and ABF2 homologous proteins were identified, with WmABF1‐1 likely functionalised akin to the ABFs in *Arabidopsis* (i.e., AtABF2, AtABF3 and AtABF4) (Figure S13a,b). Our study revealed that WmABF1‐1 could activate *WmMYB111*, shaping a transcriptional cascade to enlarge the ABA signal and promote leaf senescence through positively co‐regulating downstream genes (Figure 5, Figures S14 and S17). Meanwhile, the WmABF1‐1 independently activated these target genes (Figure S18), which highlighted the conservateness of ABF1 in promoting ABA biosynthesis, leaf senescence and chlorophyll degradation. As common activators of these genes, WmABF1‐1 and WmMYB111 interact with each other, amplifying their combined effect beyond that of WmABF1‐1/WmMYB111 alone (Figures 6 and 7i).

Leaf senescence is termed a nutrient mining and recycling process which is initiated with the development of a reproductive sink that remobilizes nutrients from vegetative tissues (Gan 2018). During this process, N is released as ${\mathrm{NH}}_{4}^{+}$ and amino acids like glutamate and aspartate from chlorophyll degradation, which are reutilized by actively growing organs (Havé et al. 2017). This regulatory mechanism enables plants to allocate their limited energy to cope with N deficiency or environmental stresses. Among the responsive phytohormones to N concentration, the synthesis of ABA has been implied to be positively correlated with high concentrations of ${\mathrm{NO}}^{3-}$ in roots (Xing et al. 2023). Conversely, exogenous ABA has inhibited leaf senescence in cucumber ( *Cucumis sativus* ) under low N conditions (Oka et al. 2012), which is similar to the enhanced NUE in wheat ears and *Brassica napus* leaves (Xie et al. 2004; Han et al. 2017). Regarding N deficiency‐induced senescence and ABA accumulation in leaves, they were found to co‐occur in rice and apple, in which MdPYL4 interacted with MdNAC4 to activate the expression of *MdNCED2* and *MdSAG39* (Zakari et al. 2020; Wen et al. 2023). These cases indicate that ABA might be tightly linked with N content fluctuations. However, rare studies have pinpointed that ABA accumulation could directly regulate the change of N content, leading to N deficiency‐induced senescence in leaves. Herein, we found N deficiency promoted leaf senescence of *Welwitschia* (Figure S19b), the sudden decrease of N content was observed in the transition from young to senescent leaf sections, and was negatively correlated with ABA levels (Figures 1c and 7a). Considering the elevated ABA level, chlorophyll degradation and N loss occurred much more severely afterwards overexpressing *WmABF1‐1* and *WmMYB111* (Figure 7b,c), we proposed that WmABF1‐1‐WmMYB111 may act as an essential regulator bridging ABA accumulation to N deficiency (Figure 8). Moreover, inasmuch as N transportation and expression of *WmNRT1.7a*/*WmNRT2.5* were strongly induced and regulated by WmABF1‐1‐WmMYB111 (Figure 7d–i, Figure S20), we further claimed that NUE in aging leaf sections could benefit from ABA‐mediated leaf senescence by improved N transportation, which is likewise to exogenous ABA treatment improving NUE in other plants (Xie et al. 2004; Han et al. 2017).

The physiological roles of ABA as a stress hormone in plant responses to water shortage have been well documented (Zhu 2016). Regarding the in situ *Welwitschia*, drought stress has considerably amplified the age‐dependent gradient in water potential in its leaves (Von Willert and Wagner‐Douglas 1994; Herppich et al. 1996). The leaf's tip was found to have the lowest water potential along the axis (Von Willert and Wagner‐Douglas 1994). Given that ABA‐induced leaf senescence could lead to an osmotic potential gradient (Zhao et al. 2016), the pattern of water potential as a whole might be closely related to the ABA content distribution in *Welwitschia* (Figure 1c). Indeed, external drought and heat could prompt ABA biosynthesis to regulate plant stomatal closure, transpiration reduction and water conservation (Zhu 2016); in turn, excessive ABA would trigger leaf senescence (Huang et al. 2022). Thus, it will be of particular interest to dissect the complex regulatory network regarding age‐dependent leaf senescence and multi‐stressed adaptation of *Welwitschia*, which can provide essential clues on addressing how external signals are integrated into the plant's internal age information.

## 4 Materials and Methods

### 4.1 Plant Materials and Growth Conditions

The *Welwitschia* individuals were ex‐situ preserved in the greenhouse of Wuhan Botanical Garden, Chinese Academy of Sciences (WBGCAS). The *Arabidopsis* wild type (Col‐0), *myb111* mutant (SALK_104164C) and *abf1* mutant (SALK_072942C) seeds were obtained from the Arabidopsis Biological Resource Center. Full‐length cDNAs of *WmNCED6*, *WmNCED9*, *WmMYB111*, *WmABF1‐1* and *AtMYB111* were cloned into the pCAMBIA‐1300‐GFP vector under the CaMV *35S* promoter to generate overexpression lines: *WmNCED6* and *WmNCED9* in Col‐0 and *myb111* mutant, *WmMYB111* in Col‐0 and *abf1* mutant, as well as *WmABF1‐1* and *AtMYB111* in Col‐0. Vectors were transformed into *Agrobacterium* cells (GV3101) and introduced into *Arabidopsis* using the flower dip method (Clough and Bent 1998). The *Arabidopsis* plants were grown in a growth chamber at 22°C under long days conditions (16:8 h light/dark photocycle). Primers used for plasmid construction are provided in Table S1.

### 4.2 Sequence Analysis

The *Welwitschia* sequences were obtained from the China National GeneBank DataBase (https://db.cngb.org/search/project/CNP0001943/). Amino acid sequences were aligned using CLUSTALW (http://www.clustal.org/clustal2/) with default parameters. The phylogenetic tree was constructed by employing the neighbour‐joining method with 1000 bootstrap replicates via MEGA (https://www.megasoftware.net/) version 6.0. Sequence specificity was verified by aligning the target sequence to whole genomes using the BLAST GUI Wrapper in TBtools software (https://tbtools.cowtransfer.com/). TF binding sites were predicted with PlantTFDB (https://planttfdb.gao‐lab.org/). The nuclear localization and protein structure were predicted using CNLS MAPPER (http://nls‐mapper.iab.keio.ac.jp/) and SWISS‐MODEL (https://swissmodel.expasy.org/), respectively.

### 4.3 RNA Extraction and RT‐qPCR

Total RNA was extracted from *Welwitschia* and *Arabidopsis* leaves using the reagent of Trizol‐Up (Invitrogen, Carlsbad, CA, USA), followed by cDNA synthesis using Superscript II (Invitrogen) mixture. For quantitative reverse transcriptase‐polymerase chain reaction (RT‐qPCR), the cDNA was diluted to 25 ng/μL as a template and mixed with 5 μL of Lightcycler FastStart DNA Master SYBR Green (Roche, Mannheim, Germany) to prepare 10 μL reaction volumes. Amplification was performed for 40 cycles on the Applied Biosystems real‐time PCR (QuantStudio 6 Flex, America Life Technologies). *WelActin* served as the internal control gene for *Welwitschia* (Moyroud et al. 2017) and *ACTIN2* for *Arabidopsis*. The detailed information of primers is provided in Table S2.

### 4.4 GUS Assay

For the β‐Glucuronidase (GUS) assay, the pMDC164‐*ProWmMYB111:GUS* vector was constructed by replacing the CaMV *35S* promoter with a 2‐kb promoter fragment upstream of the *WmMYB111* coding region. The vector was transformed into *Agrobacterium* cells (GV3101) and resuspended to an OD_600_ of 0.8 using the suspension [10 mM MgCl_2_, 10 mM 2‐(*N*‐morpholine)‐ethanesulphonic acid (MES) and 100 μM acetosyringone (AS)], and were further injected into *Welwitschia* leaves. After 3 days in the dark, the injected areas were cut out and treated with (+ABA) and without (−ABA) 10 μM ABA solution for 4 h. Then, the GUS activity assay was conducted following available protocols (Jefferson et al. 1987). Primers used for plasmid construction are provided in Table S3.

### 4.5 Subcellular Localization

The plasmids of *35S:WmNCED6‐GFP*, *35S:WmNCED9‐GFP*, *35S:WmMYB111‐GFP* and *35S:WmABF1‐1‐GFP* were transformed into *Agrobacterium* cells (GV3101) and resuspended to an OD_600_ of 0.75 with infiltration buffer (2 mM Na_3_PO_4_, 20 mM MES, 150 μM AS and 0.5% glucose). These suspensions were injected into *N. benthamiana* leaves, which were collected after 40 h. Subcellular localization was visualised using a Laser scanning confocal microscope (SP8; Leica, Wetzlar, Germany) with 488 nm (GFP) and 552 nm (RFP) excitation wavelengths. The *35s‐NLS‐DsRed* plasmid served as a nuclear marker (You et al. 2019), and primers used are provided in Table S1.

### 4.6 VIGS and Transient Transformation Assays

The specific silenced coding fragments were verified using TBtools. The fragments were inserted into the pTRV2 vector to create pTRV‐*WmNCED6*, pTRV‐*WmNCED9*, pTRV‐*WmMYB111* and pTRV‐*WmABF1‐1* constructs. The pTRV‐*CLA* (*Chloroplastos alterados 1*) vector (Zhang et al. 2023) was transiently injected into *Welwitschia* leaves to observe the visible de‐greening phenotype, serving as a marker for the activation of the pTRV2 vector. The *Agrobacterium* GV3101 cells harbouring these vectors were re‐suspended in infiltration buffer (10 mM MgCl₂, 10 mM MES and 200 μM AS) to an OD_600_ of 0.8, then were mixed with pTRV1 in equal volumes. The mixtures were cultured in the dark for 3 h at room temperature (~25°C).

For transient transformation, the green sections of *Welwitschia* leaves (S1–S5) were longitudinally bisected from base to tip. One half was used for TRV‐control and the other for gene silencing transient transformation. Each half was cut into uniform squares (5 mm × 5 mm) and submerged in bacterial suspensions of pTRV2, pTRV‐*WmNCED6*, pTRV‐*WmNCED9*, pTRV‐*WmMYB111* or pTRV‐*WmABF1‐1*. Vacuum infiltration was performed at 1.0 MPa for 5 min, with the vacuum applied and released 2–3 times until full infiltration. The leaf discs were then washed with deionised water and incubated in the dark at 8°C for 3 days. The de‐greening timing of pTRV‐*CLA*‐silenced areas was selected as the first day for phenotypic observation, and RT‐qPCR was used to assess gene silencing efficiency. Further, the leaf disc phenotypes under ABA treatment were examined using 50 μM ABA.

To quantify the senescent phenotype in transiently transformed *Welwitschia* leaf discs, the number of discs exhibiting green, green/yellow and yellow colours was recorded on days of 0th, 10th, 15th and 20th (Zhang et al. 2022). For measurements of physiological parameters such as the *F*_v_/*F*_m_ ratio, ABA content, ion leakage rate and chlorophyll content, at least 90 leaf discs were used for each transient transformation line, with three replicates per line. The same protocols were applied for gene overexpression transformations. Primer sequences for plasmid construction are provided in Table S3.

### 4.7 Quantification of Endogenous ABA Levels

Approximately 0.1 g of leaf samples were frozen and ground in liquid nitrogen. Then, 1.5 mL of 80% methanol and internal standards were added, followed by agitation at 4°C for 3 h (Zhang, Peng, et al. 2021). After centrifugation, the supernatant was re‐dissolved in 300 μL of 30% methanol, and the extracts were determined using UHPLC–MS/MS (Thermo Scientific Ultimate 3000 UHPLC coupled with TSQ Quantiva).

### 4.8 Ion Leakage Measurements

To assess the degree of electrolyte leakage in *Welwitschia* leaf discs and *Arabidopsis* leaves, the conductivity meter (ST3100C, Ohaus Corporation, Parsippany, NJ, USA) was used to measure the ion leakage rates (Yuan et al. 2020). The initial conductivity was measured after immersing the samples in deionised distilled water with gentle shaking for 2 h. The total conductivity was then determined by incubating the samples at 95°C for 15 min. Ion leakage rates were calculated as the percentage of initial conductivity relative to total conductivity. Each experiment was performed with three independent replicates.

### 4.9 Determination of Chlorophyll Contents and *F*_v_/*F*_m_ Ratios

*Welwitschia* leaf discs were frozen in liquid nitrogen for chlorophyll (Chl) extraction. Chl content was determined by measuring absorbance (Chla: A_665_, Chlb: A_649_) with a microplate reader (Infinite M200; Tecan, Mannedorf, Switzerland) (Porra et al. 1989). Sixteen leaf discs were used per experiment, with at least three biological replicates. The photochemical efficiency of photosystem II (PSII; *F*_v_/*F*_m_ ratio) in transiently transformed *Welwitschia* leaf discs and *Arabidopsis* leaves was measured using the PlantView 230F (BLT Photon Technology). All experiments included at least three biological replicates.

### 4.10 N Treatment and Determination

For N treatment, leaf discs of *Welwitschia* were placed into the Hoagland nutrient solution with different N concentrations (LN condition (NH_4_Cl [1 mM], KNO_3_ [0.94 mM], NaCl [19 mM] and KCl [17.86 mM]); NN condition (NH_4_Cl [20 mM] and KNO_3_ [18 mM]); HN condition (NH_4_Cl [40 mM] and KNO_3_ [37.6 mM])), as a previous study described (Zhang et al. 2024). For N determination, the *Welwitschia* leaves were dried at 80°C for 5 days and ground into powder. Approximately 20 mg of the powdered samples was loaded into pressed tin capsules (CHNOS; CN01251, Φ 6 × 11 mm, 0.29 mL, 200 Pcs, China), and were automatically weighed to record the dry weight (DW). The N content was measured using an automatic elemental analyser (Vario MACRO Cube, Elementar Analysensysteme GmbH, Hanau, Germany). The N content per unit leaf DW was calculated in percentages. All experiments were repeated with at least three biological replicates.

### 4.11 ChIP Assays

The *35S* EV‐control, *35S:WmMYB111*‐GFP and *35S:WmABF1‐1*‐GFP transient transformation leaf discs were used as materials for ChIP as described previously (Xu et al. 2021). The GFP antibody was used to immunoprecipitate (IP) the protein‐DNA complex. The diluted precipitated DNA served as templates for ChIP‐qPCR, and pre‐precipitation chromatin was used as the input control. The ChIP experiments were performed three times, and results are shown as relative enrichment (IP) of input DNA. All the primers used for ChIP‐qPCR are provided in Table S4.

### 4.12 Recombinant Protein Purification and EMSA

The full‐length protein of WmMYB111 and WmABF1‐1 was fused in the C‐terminal frame of MBP (pET‐28a‐MBP) and GST (pGEX‐6P‐1) then expressed in *Escherichia coli* BL21 strain, respectively. For recombinant protein induced conditions of MBP‐WmMYB111 and GST‐WmABF1‐1, 1 mM isopropylthio‐β‐D‐galactoside was used and the cultures were incubated at 16°C and 20°C for 16 h, respectively. Then, Maltose‐agarose and GST‐agarose affinity chromatography were used to purify the recombinant proteins.

For EMSA, the promoter fragments of *WmNCED6*, *WmNCED9*, *WmSEN1*, *WmSEN2*, *WmPAO1‐1*, *WmPAO2‐1*, *WmNRT1.7a*, *WmNRT2.5* that contained MBS cis‐elements and *WmMYB111*, *WmNCED6*, *WmSEN1*, *WmPAO1‐1*, *WmNRT1.7a*, *WmNRT2.5* that contained ABS cis‐elements were biotin labelled as probes. The corresponding unlabeled DNA fragments were used as competitors. Then, the EMSA assays were performed as previously described, and biotin‐labelled DNA was detected via chemiluminescence (Xu et al. 2022). Primers used in this part are listed in Table S5.

### 4.13 Y1H Assays

The promoter sequences from *WmNCED6*, *WmNCED9*, *WmSEN1*, *WmSEN2*, *WmPAO1‐1*, *WmPAO2‐1*, *WmNRT1.7a*, *WmNRT2.5* and *WmMYB111* were cloned and inserted into the pAbAi vector. After BstbI digestion, these linearized plasmids were transformed into Y1H gold strains. The aureobasidin A (AbA) gradients (0–900 ng/mL) were used in synthetic dropout medium (SD‐Ura) to assess transformed cell growth inhibition. Full‐length coding sequences of *WmMYB111* and *WmABF1‐1* were cloned into pGADT7 and transformed into yeast strains harbouring the promoter constructs. Cells were grown on SD/‐Ura/‐Leu medium with 0–900 ng/mL AbA for 3 days. Primer sequences for Y1H are provided in Table S6.

### 4.14 Dual‐Luciferase Assays

For the Dual‐luciferase assay (LUC/REN), the ORFs of *WmMYB111* and *WmABF1‐1* were attached to the pGreen II 62‐SK vector under the *35S* promoter to create effectors. Promoter fragments of *WmNCED6*, *WmNCED9*, *WmSEN1*, *WmSEN2*, *WmPAO1‐1*, *WmPAO2‐1*, *WmNRT1.7a*, *WmNRT2.5* and *WmMYB111* were cloned and inserted into pGreen II 0800‐LUC as reporter constructs. LUC/REN activity was measured using the Dual‐Luciferase Reporter Assay System (Promega) and an Infinite M200 microplate reader (Tecan, Mannedorf, Switzerland), following previously described methods (Xu et al. 2021). The in vivo imaging of luminescence was performed on infiltrated *N. benthamiana* leaves using the PlantView100 system (BLT Photon Technology). Primers used for Dual‐luciferase are provided in Table S7.

### 4.15 Y2H Assay

Full‐length coding sequences of *WmMYB111* and *WmABF1‐1* were cloned into the prey vector pGADT7 (AD‐WmMYB111 and AD‐WmABF1‐1) and bait vector pGBKT7 (BD‐WmMYB111 and BD‐WmABF1‐1), which were introduced into the Y2HGold yeast strain (Clontech). The mating experiments were carried out following available protocols (Xu et al. 2022). Primer sequences used for Y2H are provided in Table S8.

### 4.16 Co‐IP Assay

The full‐length cDNA of *WmMYB111* was inserted into the pCAMBIA‐1300‐Flag vector to construct *35S:WmMYB111‐Flag*. After co‐infiltrating *N. benthamiana* leaves with *Agrobacterium* carrying *35S:WmABF1‐1‐GFP* and *35S:WmMYB111‐Flag* (or *35S: Flag*) for 3 days, the fusion proteins were extracted using Western & IP lysis buffer (SL1000, Coolaber). The anti‐GFP antibody incubated with Protein A/G Dynabeads (Invitrogen) in advance was used to perform the immunoprecipitation (IP) with the proteins of WmABF1‐1‐GFP and WmMYB111‐Flag. The beads were collected and then resuspended in protein extraction buffer for immunoblotting (IB) analysis. Primers used are provided in Table S1.

### 4.17 BiFC Assays

The full‐length coding sequences of *WmABF1‐1* and *WmMYB111* were inserted into pYNE and pYCE vectors to generate *WmABF1‐1‐nYFP*, *WmABF1‐1‐cYFP*, *WmMYB111‐nYFP* and *WmMYB111‐cYFP*. These plasmids were transformed into *Agrobacterium* cells (GV3101) and injected into *N. benthamiana* leaves with various vector combinations (*WmABF1‐1‐nYFP* and *WmMYB111‐cYFP*, *WmABF1‐1‐cYFP* and *WmMYB111‐nYFP*, *WmABF1‐1‐nYFP* and *WmABF1‐1‐cYFP*, *WmMYB111‐nYFP* and *WmMYB111‐cYFP*, *WmABF1‐1‐nYFP* and *cYFP*, *WmABF1‐1‐cYFP* and *nYFP*, *WmMYB111‐nYFP* and *cYFP*, *WmMYB111‐cYFP* and *nYFP*, *nYFP* and *cYFP*). After 40 h, YFP fluorescence was detected using the Laser scanning confocal microscope (SP8; Leica, Wetzlar, Germany) with 488 nm excitation wavelength. Primers used for BiFC are provided in Table S8.

### 4.18 Statistical Methods

The significant differences among means from two groups were determined using two‐tailed Student's *t*‐test (*p < 0.05, **p < 0.01). The multiple range test of significant difference at *p* < 0.05 was performed using the one‐way ANOVA‐HSD test (two‐sided), which was conducted using the ‘aov’ coupled with ‘TukeyHSD’ functions (with default parameters). The R (v4.2.1; https://cran.r‐project.org/) was used for statistical analysis and plot generation.

### 4.19 Accession Numbers

The *Welwitschia* gene IDs used in this article were provided in the Table S2, from which CDS and promoter sequences can be found in the China National GeneBank DataBase (https://db.cngb.org/search/project/CNP0001943/). For *Arabidopsis* sequences, they can be found in the Arabidopsis Genome TAIR database (https://www.arabidopsis.org/) under the following accession numbers: *AtMYB111*(AT5G49330), *AtABF1*(AT1G49720), *AtNCED6*(AT3G24220), *AtNCED9*(AT1G78390), *AtSEN1*(AT4G35770), *AtPAO1*(AT5G13700), *AtPAO2*(AT2G43020), *AtSAG12*(AT5G45890) and *ACTIN2*(AT3G18780).

## Author Contributions

T.W. and Q.W. conceived and initiated the study with additional input from H.X. H.X. and T.W. designed the major scientific objectives and led the manuscript preparation together with Q.W. and J.H. H.X. has performed most of the experiments and analysed the data with the assistance from Q.S., J.W. and J.H.

## Conflicts of Interest

The authors declare no conflicts of interest.

## Supporting information


**Appendix S1:** pbi70290‐sup‐0001‐supinfo.zip.

## Data Availability

The materials and data that support the findings of this study are available on request from the corresponding author. The data are not publicly available due to privacy or ethical restrictions.
