# Supplementary material for: The Module WmABF1‐1‐WmMYB111 Manipulates the Leaf Senescence of Welwitschia by Integrative Regulation of Abscisic Acid Biosynthesis, Chlorophyll Degradation and Nitrogen Transportation
Source: Plant Biotechnol J. 2025 Aug 13;23(12):5369–86. doi: 10.1111/pbi.70290 (PMC12665073; doi:10.1111/pbi.70290)
Supplement: Supplementary file 1 — Appendix S1: pbi70290‐sup‐0001‐supinfo.zip. [file PBI-23-5369-s001.zip › 4. Supplementary Figures & Tables.docx]

**The module WmABF1-1-WmMYB111 manipulates the leaf senescence of *Welwitschia* by integrative regulation of abscisic acid biosynthesis, chlorophyll degradation and nitrogen transportation**

Xu et al.

**Supplementary Figures**

**Figure S1** The leaf senescence observed in *Welwitschia.* (a) Phenotypes of the *Welwitschia* seedlings at different DAGs (Day after germination) (up line), and the leaf senescence in *ex-situ Welwitschia* with different estimated ages. The red arrows indicated the senescence at the tip of true leaves from 30 DAG to around 12-years old individuals. (b) The leaf senescence in *in-situ* *Welwitschia.*

**Figure S2** Gene expression of *WmNCEDs*, *WmSAGs*, *WmCCGs* in leaves and characterization of *WmNCED6* and *WmNCED9*. (a) The relative expression levels of *WmNCEDs*, *WmSAGs* and *WmCCGs* in S1~S6. Data are means ± SE (*n* = 3 biological replicates), with different letters above the columns indicating the significant difference at *P* < 0.05 (one-way ANOVA-HSD test of two-sided performed with “aov" and “TukeyHSD" functions). (b) The predicted protein structure and subcellular localization of WmNCED6 (left) and WmNCED9 (right). The structure in dark blue and deep orange color indicated N-terminal and C-terminal, respectively. For subcellular localization, both *WmNCED6-GFP* and *WmNCED9-GFP* were heterologous expressed in *N. benthamiana* protoplast. Scale bar = 25 µm.

**Figure S3** Gene silencing of *WmNCED6* and *WmNCED9* in leaf discs, and the phenotype of TRV-*CLA* in injected area of *Welwitschia* leaf. (a) The selected VIGS regions and gene expression level in its silenced-discs relative to the TRV-control. Case for *WmNCED*6 (up) and *WmNCED*9 (down). Transient transformed samples used in here were collected at 0d, data are means ± SE (*n* = 3 biological replicates, each replicate contained 9~10 leaf discs). Significant difference analysis used two-tailed Student’s *t*-test (**P* < 0.05, ***P* < 0.01). (b) The phenotype of TRV-*CLA* injected area (indicated by red arrows) in ~ 3-years old *Welwitschia*, Scale bar = 1 cm.

**Figure S4** Overexpression of *WmNCED6* or *WmNCED9* could promote leaf senescence of *Welwitschia*. (a) Phenotypes of *35S*-control, *35S:WmNCED6* and *35S:WmNCED9* leaf discs in 0d, 5d, 10d and 15d, Scale bar = 5 mm. (b) Relative expression levels of *WmNCED6* and *WmNCED9* in *35S:WmNCED6* and *35S:WmNCED9* leaf discs indicated by RT-qPCR. Samples used in (b) were collected at 0d. (c) Quantification of senescent phenotypes of the leaf discs. (d-f) Physiological indices (ABA contents (d), ion leakage rates (e) and chlorophyll contents (f)) of *35S*-control, *35S:WmNCED6* and *35S:WmNCED9* leaf discs from 0d to 15d. (g) Relative expression levels of *WmSEN1*, *WmSEN*2, *WmPAO1-1* and *WmPAO2-1* in *35S*-control, *35S:WmNCED6* and *35S:WmNCED9* leaf discs indicated by RT-qPCR. All data are means ± SE (*n* = 3 biological replicates, at least 16 leaf discs were collected as each replicate). Two-tailed Student’s *t*-test (**P* < 0.05, ***P* < 0.01) were used in significant difference analysis, and ns indicates no significant difference.

**Figure S5** Overexpression of *WmNCED6* or *WmNCED9* could promote early flowering and leaf senescence in *Arabidopsis*. (a) Flowering (15 DAG) and leaf senescent (40 DAG) phenotypes of Col-0, *35S:WmNCED6#1* and *35S:WmNCED6#2*. The flowering is indicated by red arrows. Scale bar = 5 cm. (b) The expression level of *WmNCED6* relative to Col-0 in transgenic plants of *35S:WmNCED6#1* and *35S:WmNCED6#2*, respectively; The dynamic change of ABA contents and ion leakage rates are also provided. (c) The relative expression levels of *AtSEN1*, *AtPAO1*, *AtPAO2* and *AtSAG12* in Col-0, *35S:WmNCED6#1* and *35S:WmNCED6#2* leaves indicated by RT-qPCR. (d-f) Similar analysis of *WmNCED9* OE in *Arabidopsis*. For b-c and e-f, 3 individuals of Col-0 and each OE line were selected to collect leaves. Data are means ± SE (*n* = 3 biological replicates). The Student’s *t*-test (**P* < 0.05, ***P* < 0.01) of two-tailed were performed in significant difference analysis.

**Figure S6** The upstream TFs prediction of aging-induced *WmNCEDs*. The number of potential TF binding sites in the 2-kb promoters of aging-induced *WmNCEDs* were calculated. The prefix ‘*p*’ means ‘promoter’ and AT5G49330 (AtMYB111) was indicated with red arrows. The detailed TF binding motifs of each gene promoter which predicted in the website (<https://planttfdb.gao-lab.org/>) were provided in the Source Data file.

**Figure S7** The characterization of *WmMYB111*. (a) The phylogenetic tree of *Welwitschia* WmMYB111 homolog proteins (11 members) and *Arabidopsis* R2R3-MYB proteins. Bootstrap values indicate the confidence of each branch, subgroup of S1~S25 refers to the distribution of R2R3-MYBs in *Arabidopsis* (Dubos *et al*., 2010). (b) The relative expression level of *W.mirabilis.22275* in S1~S6. Data are means ± SE (*n* = 3 biological replicates), with letters of ‘a’, ‘b’ and ‘c’ above the columns indicating the significant difference at *P* < 0.05 (one-way ANOVA-HSD test of two-sided performed with “aov" and “TukeyHSD" functions). (c) The predicted protein structure of AtMYB111 and WmMYB111 (W. mirabilis.18177). (d) The conserved domain of WmMYB111 indicated by comparison of its homologs in other plants (Bs: *Boechera stricta*, Cs: *Camelina sativa*, At: *Arabidopsis thaliana*, Al: *Arabidopsis lyrata*, Cg: *Capsella grandiflora*, Cr: *Capsella rubella*, Tp: *Thellungiella parvula*, Es: *Eutrema salsugineum*, Bo: *Brassica oleracea*, Bn: *Brassica napus*, Br: *Brassica rapa*, Rr: *Raphanus raphanistrum*, Rs: *Raphanus sativus*, Si: *Sisymbrium irio*, Aa: *Arabis alpina*, Aa_2: *Aethionema arabicum*, Th: *Tarenaya hassleriana*, Cp: *Carica papaya*, Cs_2: *Citrus sinensis*, Cc: *Citrus clementina*, Wm: *Welwitschia mirabilis*). The two conserved SANT domains are highlighted by green rectangle. (e) NLS prediction (red sequence) and subcellular localization of WmMYB111. The *WmMYB111-GFP* was heterologous expressed in *N. benthamiana* protoplast which indicated by yellow rectangle. m-Cherry was used as the marker to indicate the nuclei. Scale bar = 10 µm. (f) GUS assay of the transient-transformed *Welwitschia* leaf discs of *ProWmMYB111:GUS* without (-) and with (+) ABA treatment. Scale bar = 5mm.

**Figure S8** Silencing of *WmMYB111* in *Welwitschia* leaf discs and the consequence after ABA treatment. (a) The *WmMYB111* silenced-region selection and its expression level in TRV-control and TRV-*WmMYB111*-silenced leaf discs. Samples were collected at 0d. (b) The phenotypic analysis, ion leakage rates and chlorophyll contents of TRV-control and TRV-*WmMYB111*-silenced leaf discs at 3d under the condition of ABA (50µM) treatment, Scale bar = 5 mm. Data are means ± SE (*n* = 3 biological replicates, each replicate included 9~10 leaf discs). Two-tailed Student’s *t*-test (**P* < 0.05, ***P* < 0.01) were conducted.

**Figure S9** Overexpression of *WmMYB111* could promote leaf senescence of *Welwitschia*. (a) Phenotypes of *35S*-control and *35S:WmMYB111* leaf discs in 0d, 5d, 10d and 15d, Scale bar = 5 mm. (b) The relative expression level of *WmMYB111* in *35S*-control and *35S:WmMYB111* leaf discs indicated by RT-qPCR. Samples were collected at 0d. (c) Quantification of senescent phenotypes of the leaf discs shown in (a). (d-f) Physiological indices (ABA contents (d), ion leakage rates (e) and chlorophyll contents (f)) of *35S*-control and *35S:WmMYB111* leaf discs from 0d to 15d. (g) The relative expression levels of *WmNCED6*, *WmNCED9*, *WmSEN1*, *WmSEN*2, *WmPAO1-1* and *WmPAO2-1* in *35S*-control and *35S:WmMYB111* leaf discs by RT-qPCR. The above data are means ± SE (*n* = 3 biological replicates, with ≥ 16 leaf discs as each replicate). Student’s *t*-test (**P* < 0.05, ***P* < 0.01) of two-tailed were applied in significant difference analysis, and ns indicates no significant difference.

**Figure S10** Overexpression of *WmMYB111* or *AtMYB111* could promote early flowering and leaf senescence of *Arabidopsis*. (a) Flowering (15 DAG) and leaf senescent (40 DAG) phenotypes of Col-0, *35S:WmMYB111#1* and *35S:WmMYB111#2*, Scale bar = 5 cm. (b) The expression level of *WmMYB111* relative to Col-0 in *WmMYB111*-OE lines (*35S:WmMYB111#1* and *35S:WmMYB111#2*), respectively; The dynamic change of ABA contents and ion leakage rates are also provided. (c) The relative expression levels of *AtSEN1*, *AtPAO1*, *AtPAO2*, *AtNCED6*, *AtNCED9* and *AtSAG12* in Col-0 and *WmMYB111*-OE lines by RT-qPCR. (d) Expression pattern of *AtMYB111* shown in eFP website (<https://www.bar.utoronto.ca/efp2/>). (e) Leaf senescent (40 DAG) phenotypes and gene expression analysis (*AtMYB111* and *AtSAG12*) of Col-0, *35S:AtMYB111#1* and *35S:AtMYB111#2*, Scale bar = 5 cm. For Col-0 and each OE line, 3 individuals were selected to check. Data are means ± SE (*n* = 3 biological replicates), with the two-tailed Student’s *t*-test (**P* < 0.05, ***P* < 0.01).

**Figure S11** Overexpression of *WmNCED6* or *WmNCED9* could promote leaf senescence of *myb111* mutant. (a) Leaf senescent (35 DAG) phenotypes of *myb111*, *35S:WmNCED6/myb111#1*, *35S:WmNCED6/myb111#2* and *F*_v_/*F*_m_ ratios in the third and fourth rosette leaves, as well as gene expression level of *WmNCED6*. Scale bar = 5 cm. (b) *F*_v_/*F*_m_ ratio, ABA contents and ion leakage rates of leaves shown in (a). (c) The relative expression levels of *AtSEN1*, *AtPAO1*, *AtPAO2* and *AtSAG12* in *myb111*, *35S:WmNCED6/myb111#1* and *35S:WmNCED6/myb111#2* leaves checked by RT-qPCR. (d-f) Similar analysis of *WmNCED9* OE in *myb111* mutant. Three individuals of *myb111*, *WmNCED6*-OE/*myb111* and *WmNCED9*-OE/*myb111* lines were analyzed, data are means ± SE (*n* = 3 biological replicates). Student’s *t*-test (**P* < 0.05, ***P* < 0.01) with two-tailed were used in all significant difference analysis.

**Figure S12** WmMYB111 binds to and activates the promoter of its target genes. (a) Y1H assays showing interactions between WmMYB111 and promoter fragments of target genes. Interactions between bait and prey were determined by cell growth on synthetic dropout medium lacking both of Ura and Leu (SD/-Ura/-Leu), which containing 0 ng·mL^-1^, 300 ng·mL^-1^ and 500 ng·mL^-1^ AbA, respectively. (b) Images of dual-luciferase assays conducted in leaves of *N. benthamiana* with different combinations of *WmMYB111* and the promoters of its target genes. The range of luminescence: 1.776e+5 ~ 4.310e+5. Data are means ± SE (*n* = 3), with different letters above the columns showing the significant difference at *P* < 0.05 (one-way ANOVA-HSD test of two-sided performed).

**Figure S13** The phylogenetic relationship of WmABIs and WmABFs and characterization of *WmABF1-1*. (a) The phylogenetic relationship of *W. mirabilis* WmABI3 paralogs and *W. mirabilis* WmABF1, which were inferred by using MEGA 6.0. Bootstrap values indicate the confidence of each branch. (b) Relative expression levels of *WmABI3*, *WmABF1* and *WmABF2* in six internal leaf sections of *Welwitschia*. (c) The relative expression level of *WmABF1-1* with ABA treatment. Data are means ± SE (*n* = 3 biological replicates). Different letters above the columns of each compartment indicate a significant difference at *P* < 0.05 (one-way ANOVA-HSD test of two-sided driven by “aov" coupled with “TukeyHSD" functions). (d) The predicted protein structures of AtABF1 and WmABF1-1. The structure in red and blue color indicated N-terminal and C-terminal, respectively. (e) NLS prediction of WmABF1-1 (red sequence). (f) Subcellular localization of heterologous expressed *WmABF1-1-GFP* in *N. benthamiana* leaf. m-Cherry was used as the marker to indicate the nuclei. Scale bar = 100 µm.

**Figure S14** The regulation of WmABF1-1 to *WmMYB111*. (a) Y1H assays showing interactions between WmABF1-1 and promoter fragments of *WmMYB111*. Interactions between bait and prey were determined by cell growth on synthetic dropout medium lacking both of Ura and Leu (SD/-Ura/-Leu), which containing 0 ng·mL^-1^, 200 ng·mL^-1^ and 700 ng·mL^-1^ AbA, respectively. (b) Images of dual-luciferase assays conducted in leaves of *N. benthamiana* with different combinations of *WmABF1-1* and *WmMYB111* promoter. The range of luminescence: 1.533e+5 ~ 5.732e+5. Data are means ± SE (*n* = 3), with letters above the columns indicating the significant difference at *P*<0.05 (one-way ANOVA-HSD test of two-sided performed with “aov" and “TukeyHSD" functions).

**Figure S15** Silencing of *WmABF1-1* in *Welwitschia* leaf discs and the consequence after ABA treatment, as well as its overexpression. (a) The selected VIGS region of *WmABF1-1* and its expression level in TRV-control and TRV-*WmABF1-1*-silenced leaf discs. (b) The phenotypic analysis, ion leakage rates and chlorophyll contents of TRV-control and TRV-*WmABF1-1*-silenced leaf discs at 3d after treatment of ABA (50µM), Scale bar = 5 mm. (c) Relative expression level of *WmABF1-1* in *35S*-control and *35S:WmABF1-1* leaf discs indicated by RT-qPCR. For (a) and (c), the samples were collected at 0d. All the data are means ± SE (*n* = 3 biological replicates, more than 9 leaf discs were used as a replicate). Two-tailed Student’s *t*-test (**P* < 0.05, ***P* < 0.01) were performed.

**Figure S16** Overexpression of *WmABF1-1* could promote leaf senescence of *Welwitschia*. (a) Senescent phenotypes and *F*_v_/*F*_m_ ratios of *35S*-control and *35S:WmABF1-1* leaf discs in 0d, 5d, 10d, 15d and 20d, Scale bar = 5 mm. (b) Quantification of senescent phenotypes of the leaf discs as shown in (a). (c) The *F*_v_/*F*_m_ ratio of leaves shown in (a). (d) The dynamic change of ABA contents. (e) The dynamic change of ion leakage rates. (f) Relative expression levels of *WmMYB111*, *WmNCED6*, *WmNCED9*, *WmSEN1*, *WmSEN*2, *WmPAO1-1* and *WmPAO2-1* in *35S*-control and *35S:WmABF1-1* leaf discs indicated by RT-qPCR. All data are means ± SE (*n* = 3 biological replicates, more than 16 leaf discs were used as each replicate). Two-tailed Student’s *t*-test (**P* < 0.05, ***P* < 0.01) were used in significant difference analysis, and ns indicates no significant difference.

**Figure S1****7** Overexpression of *WmABF1-1* and *WmMYB111* promotes early flowering and leaf senescence of Col-0 and *abf1* mutant, respectively. (a) Flowering (15 DAG) and leaf senescent (40 DAG) phenotypes of Col-0, *35S:WmABF1-1#1* and *35S:WmABF1-1#2*, Scale bar = 5 cm. (b) The expression levels of *WmABF1-1* and *AtSAG12* relative to Col-0 in *WmABF1-1*-OE lines, respectively. (c) Expression pattern of *AtABF1* (AT1G49720) in leaf shown in eFP website (<https://www.bar.utoronto.ca/efp2/>). (d-e) *WmMYB111* OE promotes early flowering and leaf senescence of *abf1* mutant, accompanied with highly *AtSAG12* expressed. For Col-0, *abf1* mutant and each OE line, 3 individuals were selected to check, data are means ± SE (*n* = 3 biological replicates), with the two-tailed Student’s *t*-test (**P* < 0.05, ***P* < 0.01).

**Figure S18** WmABF1-1 activates the expression of *WmNCED6*, *WmSEN1* and *WmPAO1-1* by binding to their promoters. (a) The schematic of *WmNCED6*, *WmNCED9*, *WmSEN1*, *WmSEN*2, *WmPAO1-1* and *WmPAO2-1* promoter regions containing ABS clusters. The candidate ABS (CACGTGGC) are highlighted in red. The number and relative position of ABS in respective promoters relative to the ATG start codon are indicated here. The prefix ‘‘*p*’’ means the ‘promoter’. The A: −600 ~ −326 bp, B: −1486 ~ −1202 bp and C: −1657 ~ −1377 bp which contain ABS were the fragments used in dual-luciferase assays shown in (c). (b) ChIP-qPCR analysis of WmABF1-1 protein enrichment in motifs of *pWmNCED6*-ABS4, *pWmSEN1*-ABS1 and *pWmPAO1-1*-ABS1. Data are means ± SE (*n* = 3), with *p*-values calculated by two-tailed Student’s *t*-test (**P* < 0.05, ***P* < 0.01). (c) Diagram of the constructs used in the dual-luciferase assays and the regulation of WmABF1-1 on *pWmNCED6, pWmSEN1 and pWmPAO1-1* (up panel). The images of dual-luciferase assays conducted in leaves of *N. benthamiana* with different combinations of *WmABF1-1* with *pWmNCED6, pWmSEN1 and pWmPAO1-1* (down panel). The range of luminescence: 1.963e+5 ~ 4.292e+5.

**Figure S19** *Welwitschia* N transport associated gene expression levels in S1~S6 and leaf senescent phenotypes in different N concentration. (a) The relative expression levels of *WmNRTs* and *WmNIAs* in *Welwitschia* six defined internal leaf sections (S1~S6). Data are means ± SE (*n* = 3 biological replicates). Different letters above the columns of each compartment indicate a significant difference at *P* < 0.05 (one-way ANOVA-HSD test of two-sided conducted by “aov" and “TukeyHSD" functions). (b) Phenotypes of *Welwitschia* leaf discs (15d) under different N conditions (LN, low N; NN, normal N; HN, high N), Scale bar = 5 mm.

**Figure S20** Overexpression of *WmABF1-1* or *WmMYB111* promotes *Arabidopsis* nitrogen transport from leaf to seed and activates promoters of *WmNRT1.7a* and *WmNRT2.5*. (a) The nitrogen content in leaves and seeds of Col-0, *35S:WmMYB111#1*, *35S:WmABF1-1#2*, *abf1* mutant and *35S:WmMYB111/abf1#1* plants. Data are means ± SE (*n* = 3 biological replicates). Different letters above the columns of each compartment indicate a significant difference at *P* < 0.05 (one-way ANOVA-HSD test of two-sided conducted by “aov" and “TukeyHSD" functions). (b) Diagram of constructs used in dual-luciferase assays, showing WmABF1-1/WmMYB111 regulation on *WmNRT1.7a* and *WmNRT2.5*. Images of dual-luciferase assays conducted in leaves of *N. benthamiana* with different combinations of *WmABF1-1*/*WmMYB111* and *pWmNRT1.7a* (P1 and P2)/*pWmNRT2.5* (P1 and P3). The range of luminescence: 1.082e+6 ~ 2.427e+6.

**Supplementary Tables**

| **Supplemental Table S1. Primers used for gene clone and OE vector construction** | |
| --- | --- |
| **Primer name** | **Sequence** |
| *WmNCED6-*full*-*F: | 5'-ATGTTCGCCGCGAGACACT-3' |
| *WmNCED6-*full-R: | 5'-TCAAGCGGCGTGGATCAT-3' |
| *WmNCED6-GFP-*F: | 5'-GAGCTCGGTACCCGGGGATCCATGTTCGCCGCGAGACACT-3' |
| *WmNCED6-GFP-*R: | 5'-GCCCTTGCTCACCATGTCGACAGCGGCGTGGATCATGCC-3' |
| *WmNCED9-*full*-*F: | 5'-ATGTCCATGGCTTTAACGAGCA-3' |
| *WmNCED9-*full*-*R: | 5'-TTATACAGGGGGACGTTGCTG-3' |
| *WmNCED9-GFP-*F: | 5'-GAGCTCGGTACCCGGGGATCCATGTCCATGGCTTTAACGAGCA-3' |
| *WmNCED9-GFP-*R: | 5'-GCCCTTGCTCACCATGTCGACTACAGGGGGACGTTGCTGC-3' |
| *WmMYB111-*full*-*F: | 5'-ATGGGAAGAGCGCCGCGA-3' |
| *WmMYB111-*full*-*R: | 5'-TTAGTAGTTCAGATAAGGAAATAGTCTGTCA-3' |
| *WmMYB111-GFP-*F: | 5'-GAGAACACGGGGGACGAGCTCATGGGAAGAGCGCCGCGA-3' |
| *WmMYB111-GFP-*R: | 5'-CATGTCGACTCTAGAGGATCCGTAGTTCAGATAAGGAAATAGTCTGTCATAA-3' |
| *WmMYB111-Flag-*F: | 5'-GACACTAGTGGATCCAAAGAATTCATGGGAAGAGCGCCGCGA-3' |
| *WmMYB111-Flag-*R: | 5'-TCCCTCGAGAAGCTTTTTGAATTCGTAGTTCAGATAAGGAAATAGTCTGTCATAA-3' |
| *AtMYB111-GFP-*F: | 5'-GAGAACACGGGGGACGAGCTCATGGGAAGGGCTCCGTGTT-3' |
| *AtMYB111-GFP-*R: | 5'-CATGTCGACTCTAGAGGATCCCTCCAATGTTATCTCTCCAATATC-3' |
| *WmABF1-1-*full*-*F: | 5'-ATGGTTTCGCAGACAGAGTCAAG-3' |
| *WmABF1-1-*full*-*R: | 5'-TCACCAAGGACCTGTTAAGGTTC-3' |
| *WmABF1-1-GFP-*F: | 5'-GAGAACACGGGGGACGAGCTCATGGTTTCGCAGACAGAGTCAAG-3' |
| *WmABF1-1-GFP-*R: | 5'-CATGTCGACTCTAGAGGATCCCCAAGGACCTGTTAAGGTTC-3' |
|  |  |

| **Supplemental Table S2. Primers used for RT-qPCR** | |
| --- | --- |
| **Primer name** | **Sequence** |
| ***WmNCEDs*:** |  |
| *W.mirabilis.03482 (WmNCED3)*-F: | 5'-AACCGCTGGAAAACTACTTGCT-3' |
| *W.mirabilis.03482 (WmNCED3)*-R: | 5'-CCACAAATGGAACCAGAAGCA-3' |
| *W.mirabilis.16164 (WmNCED4-1)*-F: | 5'-GATTAAAAATAGAGATCGCGGGA-3' |
| *W.mirabilis.16164 (WmNCED4-1)*-R: | 5'-ATCCACTTATGTCTCTGTTGCTGC-3' |
| *W.mirabilis.26156 (WmNCED4-2)*-F: | 5'-TCCGTTGAGTTGCCGTCG-3' |
| *W.mirabilis.26156 (WmNCED4-2)*-R: | 5'-CCCGCCTTCCCGCAC-3' |
| *W.mirabilis.26157 (WmNCED4-3)*-F: | 5'-CCAGGCACCGCCCAAG-3' |
| *W.mirabilis.26157 (WmNCED4-3)*-R: | 5'-TTACGGACGAAGAATCGCTGT-3' |
| *W.mirabilis.07750 (WmNCED6)*-F: | 5'-TGTTCGCCGCGAGACACT-3' |
| *W.mirabilis.07750 (WmNCED6)*-R: | 5'-GAGCCGAGAATAAATAGCAGACG-3' |
| *W.mirabilis.24442 (WmCCD7)*-F: | 5'-GCAAAGAAACTTGATAAAGGAGAGG-3' |
| *W.mirabilis.24442 (WmCCD7)*-R: | 5'-GCAGATGCCCAAAAGCCA-3' |
| *W.mirabilis.08267 (WmCCD8)*-F: | 5'-ATGTTGTGCTTTTGGATGGTGA-3' |
| *W.mirabilis.08267 (WmCCD8)*-R: | 5'-AATTTATTAGGAACCCAACAACCAT-3' |
| *W.mirabilis.11040 (WmNCED9)*-F: | 5'-ATGCGGCTTCTCCAAATCTCT-3' |
| *W.mirabilis.11040 (WmNCED9)*-R: | 5'-ACGAAGGTGCCGTGAAAGC-3' |
| ***WmSAGs*:** |  |
| *W.mirabilis.02705 (WmSEN1)*-F: | 5'-CTAGTTGTTGGGTGCCAAAGTG-3' |
| *W.mirabilis.02705 (WmSEN1)*-R: | 5'-CACCTCCCACGTCAGTTATCTTAG-3' |
| *W.mirabilis.13186 (WmSEN2)*-F: | 5'- ACCGAAAGAAAACAACTTTAAACAAGCA-3' |
| *W.mirabilis.13186 (WmSEN2)*-R: | 5'- AGAGCGTATCTCTGCTGAAACT-3' |
| *W.mirabilis.12484 (WmSEN4)*-F: | 5'-AGCATGGATAAAACGTCTGGTG-3' |
| *W.mirabilis.12484 (WmSEN4)*-R: | 5'-TCTGCTCCCTGTTTCCAATTC-3' |
| *W.mirabilis.10040 (WmSAG2)*-F: | 5'-AACTCCTGGGGAGAATCATGG-3' |
| *W.mirabilis.10040 (WmSAG2)*-R: | 5'-GAACAATTGGATAGGATGCACAAG-3' |
| *W.mirabilis.09351 (WmSAG20)*-F: | 5'-CTGCTTCTGATTCTGATGCTCG-3' |
| *W.mirabilis.09351 (WmSAG20)*-R: | 5'-GATGGCAAGAACAAGGCCAG-3' |
| *W.mirabilis.01220 (WmSAG24)*-F: | 5'-ATTCTTCCAGACGGTGTAAATGC-3' |
| *W.mirabilis.01220 (WmSAG24)*-R: | 5'-TGCAATGCTGGAGACAAGAAAG-3' |
| *W.mirabilis.21642 (WmSAG101)*-F: | 5'-TAGTTTTATGCAATGGTGGAATCAG-3' |
| *W.mirabilis.21642 (WmSAG101)*-R: | 5'-TGCCCTCTATCATTTGGTAAAGC-3' |
| *W.mirabilis.03214 (WmSAG113)*-F: | 5'-ATGGAAGAGGTGGAGTTTTGTTG-3' |
| *W.mirabilis.03214 (WmSAG113)*-R: | 5'-CCGAGCCGCGCGAG-3' |
| ***WmCCGs*:** |  |
| *W.mirabilis.03320 (WmNYE1)*-F: | 5'-CGAGAGCCGGAAATCGGT-3' |
| *W.mirabilis.03320 (WmNYE1)*-R: | 5'-CGGACTTTTTGTTCAACTGCCT-3' |
| *W.mirabilis.08028 (WmNYE2)*-F: | 5'-CAAGGGTCAGCCCAAGAAGA-3' |
| *W.mirabilis.08028 (WmNYE2)*-R: | 5'-CGAACGGTGGAAAACAACAGT-3' |
| *W.mirabilis.18758 (WmPAO1-1)*-F: | 5'-CACGTCTTCCACATGGGAAAC-3' |
| *W.mirabilis.18758 (WmPAO1-1)*-R: | 5'-TTTCTGGGCTTTTCTCCTCG-3' |
| *W.mirabilis.18757 (WmPAO1-2)*-F: | 5'-CTCGTTTACCCTGTTTCTTCCCT-3' |
| *W.mirabilis.18757 (WmPAO1-2)*-R: | 5'-ACGATAATGGTGCTCATCTCCTG-3' |
| *W.mirabilis.18756 (WmPAO1-3)*-F: | 5'-TTTCTAGCTCGGGAATAGAACGA-3' |
| *W.mirabilis.18756 (WmPAO1-3)*-R: | 5'-CATTTTGTAAGGTCCACCGTTTC-3' |
| *W.mirabilis.11075 (WmPAO2-1)*-F: | 5'-TGCAGAAGATATTTCCTTCAAAGGT-3' |
| *W.mirabilis.11075 (WmPAO2-1)*-R: | 5'-GTGGCGCAGATGCATGTGTA-3' |
| *W.mirabilis.06160 (WmPAO2-2)*-F: | 5'-AAAGCCAATGCAATTGCATTTGAGC-3' |
| *W.mirabilis.06160 (WmPAO2-2)*-R: | 5'-CATATGTAGTAGGTGCGACC-3' |
| *W.mirabilis.11558 (WmPAO5)*-F: | 5'-CCATCGTCCCTCCTTTACAGC-3' |
| *W.mirabilis.11558 (WmPAO5)*-R: | 5'-TGCTGAAGGAGACGATTGGC-3' |
| ***WmMYB111s*:** |  |
| *W.mirabilis.18177 (WmMYB111)-*F: | 5'-AGATCGAGTCTTGCGTGGTTTAC-3' |
| *W.mirabilis.18177 (WmMYB111)-*R: | 5'-AGGAAATAGTCTGTCATAATCGTAGCA-3' |
| *W.mirabilis.22275-*F: | 5'-ACAAATGCGAGTCGGAAGG-3' |
| *W.mirabilis.22275-*R: | 5'-GACCTTCCACATCCAGAAAATC-3' |
| ***WmABIs*:** |  |
| *W.mirabilis.20329 (WmABI3-1)-*F: | 5'-TCGGATGCTCTTAACTTTTCC-3' |
| *W.mirabilis.20329 (WmABI3-1)-*R: | 5'-TTCAAGTTCTTCTCCGGCTTC-3' |
| *W.mirabilis.06123 (WmABI3-2)-*F: | 5'-AGTTTCCGGCACAGAAGG-3' |
| *W.mirabilis.06123 (WmABI3-2)-*R: | 5'-CCAAAAGAAAAATACCCAG-3' |
| *W.mirabilis.14142 (WmABI3-3)-*F: | 5'-CAGGAAAGAAAACCCAAGAATTTAAAG-3' |
| *W.mirabilis.14142 (WmABI3-3)-*R: | 5'-TCTTGCAGTTAGCTCTGGCA-3' |
| *W.mirabilis.18424 (WmABI3-4)-*F: | 5'-GCGCAGAAACATTGCAGAG-3' |
| *W.mirabilis.18424 (WmABI3-4)-*R: | 5'-TTTTGTAAGTACCTTGGCGACG-3' |
| *W.mirabilis.08317 (WmABI3-5)-*F: | 5'-AACAACAAAACCAGAATGTATGTATTG-3' |
| *W.mirabilis.08317 (WmABI3-5)-*R: | 5'-GCTCAGCTGATACGTCTTTTC-3' |
| ***WmABFs*:** |  |
| *W.mirabilis.09338 (WmABF1-1)-*F: | 5'-ATGTATGGCGCGAGATTCATAAGG-3' |
| *W.mirabilis.09338 (WmABF1-1)-*R: | 5'-CGATCTGGTTCTTCTCTAACCAC-3' |
| *W.mirabilis.26714 (WmABF1-2)-*F: | 5'-TATTCGTTGACGTTGGAGGAATTC-3' |
| *W.mirabilis.26714 (WmABF1-2)-*R: | 5'-TCCGCAGTCCAAATGTTCTTC-3' |
| *W.mirabilis.20684 (WmABF1-3)-*F: | 5'-CGCATGATAAAGAACCGCGA-3' |
| *W.mirabilis.20684 (WmABF1-3)-*R: | 5'-CTGAGTTGCATAACCTCGTTC-3' |
| *W.mirabilis.25131 (WmABF2)-*F: | 5'-TGAAAGAGGAGAATGTCGATCC-3' |
| *W.mirabilis.25131 (WmABF2)-*R: | 5'-TGCCTGATCTCTCTAGACAG-3' |
| ***WmNRTs*:** |  |
| *W.mirabilis.24023 (WmNRT1.1a)-*F: | 5'-AGTGCAATCATGTGCGCAGT-3' |
| *W.mirabilis.24023 (WmNRT1.1a)-*R: | 5'-CATGAAGAAGCAAAGGGTCTG-3' |
| *W.mirabilis.09556 (WmNRT1.1b)-*F: | 5'-CATGCAGCAACCACCATGAAT-3' |
| *W.mirabilis.09556 (WmNRT1.1b)-*R: | 5'-GAAACGGTTAAAGAAGGAGTCC-3' |
| *W.mirabilis.08641 (WmNRT1.5a)-*F: | 5'-ACGCAGGTGGAAGAGGTG-3' |
| *W.mirabilis.08641 (WmNRT1.5a)-*R: | 5'-GCGACGCTAATTATGTCGAAC-3' |
| *W.mirabilis.23524 (WmNRT1.5b)-*F: | 5'-CTTGTCCTCGTGAACCAAGG-3' |
| *W.mirabilis.23524 (WmNRT1.5b)-*R: | 5'-CGCCGAGCAAAGAGAATATG-3' |
| *W.mirabilis.05778 (WmNRT1.7a)-*F: | 5'-TTAAGGCCACTGGAGTGCAAAA-3' |
| *W.mirabilis.05778 (WmNRT1.7a)-*R: | 5'-AAGGTTTGTATGTGCTCTCTGC-3' |
| *W.mirabilis.12362 (WmNRT1.7b)-*F: | 5'-TTACTGCTCTCACTCTCGTG-3' |
| *W.mirabilis.12362 (WmNRT1.7b)-*R: | 5'-CTGCCCATTTCCAAAATCTGC-3' |
| *W.mirabilis.13106 (WmNRT2.5)-*F: | 5'-GTATCCGCGGCGCTGTGCA-3' |
| *W.mirabilis.13106 (WmNRT2.5)-*R: | 5'-AAGTATTCGGCTATGACGTTGTCGAC-3' |
| *W.mirabilis.09029 (WmNRT3.1a)-*F: | 5'-CCTGGAGGTGCAGGCGAGT-3' |
| *W.mirabilis.09029 (WmNRT3.1a)-*R: | 5'-ACAGAGCTTCAGACTCAGAGTACTG-3' |
| *W.mirabilis.04517 (WmNRT3.1b)-*F: | 5'-CGCGTGCCTCGTGAAGAC-3' |
| *W.mirabilis.04517 (WmNRT3.1b)-*R: | 5'-ACGCTCCATGTCACTTTTCAGC-3' |
| *W.mirabilis.08116 (WmNIA1a)-*F: | 5'-GGGGCCGTATGGGCAAATC-3' |
| *W.mirabilis.08116 (WmNIA1a)-*R: | 5'-TGCGGTTAGCGTACACAAGG-3' |
| *W.mirabilis.03576 (WmNIA1b)-*F: | 5'-GAAAGCACTGGTGCTGGTG-3' |
| *W.mirabilis.03576 (WmNIA1b)-*R: | 5'-CGATACCAGCAGTTGTTCATC-3' |
| ***Actin* in *Welwitschia*:** |  |
| *W.mirabilis.10790 (WelActin)-*F: | 5'-TTGCAATTCAGGCAGTTTTG-3' |
| *W.mirabilis.10790 (WelActin)-*R: | 5'-GGCCACATATGCCAACTTCT-3' |
| ***Arabidopsis* genes:** |  |
| *AtNCED6-*F*:* | 5'-AGCTCGACTCGCTCTCTTCAC-3' |
| *AtNCED6-*R*:* | 5'-GCCGTTAAAGAAAACCACACC-3' |
| *AtNCED9-*F*:* | 5'-AACCAATACCAAAACCCCAAAC-3' |
| *AtNCED9-*R*:* | 5'-TCGAAGAGGAAGATGAAGTTGAG-3' |
| *AtPAO1-*F*:* | 5’-GCTCTAACATTCTGGTGGTGACG-3’ |
| *AtPAO1-*R*:* | 5’-CCCAAACATGTCCCTGAGAACAC-3’ |
| *AtPAO2-*F*:* | 5’-AGATTGTAGGATGCGAGTTC-3’ |
| *AtPAO2-*R*:* | 5’-TTAGACGATATAAGAAGAGG-3’ |
| *AtSEN1-*F: | 5’-CTGGTCATCGGCTATTTCTCC-3’ |
| *AtSEN1-*R: | 5’-CTGCTTGTTGTCGTTGCTTTC-3’ |
| *AtACTIN2-*F: | 5'-TGTGCCAATCTACGAGGGTTT-3' |
| *AtACTIN2-*R: | 5'-TTTCCCGCTCTGCTGTTGT-3' |

| **Supplemental Table S3. Primers used for VIGS and GUS assay** | |
| --- | --- |
| **Primer name** | **Sequence** |
| TRV-*WmNCED6-*F: | 5'-GGGGTACCATGTTCGCCGCGAGACACT-3' |
| TRV-*WmNCED6-*R: | 5'-CCGCTCGAGGAGCCGAGAATAAATAGCAGAC-3' |
| TRV-*WmNCED9-*F: | 5'-GGGGTACCGATGCGGCTTCTCCAAATCT-3' |
| TRV-*WmNCED9-*R: | 5'-CCGCTCGAGGACGAAGGTGCCGTGAAAG-3' |
| TRV-*WmMYB111-*F: | 5'-GGGGTACCATGGGAAGAGCGCCGCGA-3' |
| TRV-*WmMYB111-*R: | 5'-CCGCTCGAGGCAGAGAATATCATCTTCTTC-3' |
| TRV-*WmABF1-1-*F: | 5'-GGGGTACCGTCTGGCAAGGCAGACGA-3' |
| TRV-*WmABF1-1-*R: | 5'-CCGCTCGAGCCAAATCAAAAGGCTGCCCAT-3' |
| *pWmMYB111:GUS-*F: | 5'-CCCGGGGATCGATCCTCTAGACCCTTAATTAATCCTTTTTCATCTTTT-3' |
| *pWmMYB111:GUS-*R: | 5'-AGTGCCAAGCTCTAGTTAATTAATCTGTACGTCCTTTAGCCCCTC-3' |
|  |  |

| **Supplemental Table S4. Primers used for ChIP-qPCR** | |
| --- | --- |
| **Primer name** | **Sequence** |
| *pWmNCED6*-MBS2/ABS4-F: | 5'-GGACGGACGGTGGAAAGC-3' |
| *pWmNCED6*-MBS2/ABS4-R: | 5'-TACCCGGTATTTGTTCATTTGAAG-3' |
| *pWmNCED9*-MBS-F: | 5'-TGGATTACGGATATCGACTTCAAC-3' |
| *pWmNCED9*-MBS-R: | 5'-ATTATTAAAACGTTCGTCTTACTATGACC-3' |
| *pWmSEN1*-MBS2/ABS1-F: | 5'-GGAAACATTTACATCCAGATATGCTT-3' |
| *pWmSEN1*-MBS2/ABS1-R: | 5'-TGAAAGGACTGACCTTATGCTTTC-3' |
| *pWmSEN2*-MBS2-F: | 5'-TCTGCACAATTCAAATTCTACACATC-3' |
| *pWmSEN2*-MBS2-R: | 5'-TGGAAGATGAATGTCCGATCAA-3' |
| *pWmPAO1-1*-MBS1/ABS1-F: | 5'-AAAGAGAATTAAAAAGTAATCTAGTAAAATGG-3' |
| *pWmPAO1-1*-MBS1/ABS1-R: | 5'-CAATCGCCTTTGTCAATTCGTA-3' |
| *pWmPAO2-1*-MBS3,4-F: | 5'-GATTGGAAATTCAATTTTATTCATTCTT-3' |
| *pWmPAO2-1*-MBS3,4-R: | 5'-CATCCTTGTGATAAATGGGACATC-3' |
| *pWmNRT1.7a*-P1-F: | 5'-GTTAATTGTTGAATATAAATTTTTAAATTGAGT-3' |
| *pWmNRT1.7a*-P1-R: | 5'-GTGCCTTAGTAGCTTTGAGTTTCTTC-3' |
| *pWmNRT2.5*-P2-F: | 5'-AGCATATAGATTTAGTGGTAAGGCATG-3' |
| *pWmNRT2.5*-P2-R: | 5'-AGCCTCTGTTCTTTTGCCTTCTAC-3' |
| *pWmNRT2.5*-P3-F: | 5'-GGTCTTCGCCATGGCGAC-3' |
| *pWmNRT2.5*-P3-R: | 5'-GGAGCTCATCCAGAACTGACAAC-3' |
| *pWmMYB111*-ABS2-F: | 5'-CATGCAACAAGCTCACTTGGAT-3' |
| *pWmMYB111*-ABS2-R: | 5'-CTTTAATGGAATGTCTTGTTTTCCA-3' |
| *pWmMYB111*-ABS3-F: | 5'-GAGGATCTAGGCCATTTTTTTCAC-3' |
| *pWmMYB111*-ABS3-R: | 5'-TTCCAATTATGTTATTTTGCTCCTTAT-3' |
| *pWmMYB111*-ABS4-F: | 5'-GCACACTGAATAACCTACAAAAACTAGA-3' |
| *pWmMYB111*-ABS4-R: | 5'-GGTGCGTTAACTCTGTGGTGTG-3' |
| *pWelActin*-F: | 5'-CTCAGCACCTTCCAGCAGGT-3' |
| *pWelActin*-R: | 5'-GACGGACCAGATTCATCGTATTC-3' |
|  |  |

| **Supplemental Table S5. Primers used for recombinant protein purification and EMSA** | |
| --- | --- |
| **Primer name** | **Sequence** |
| pET-28a-*WmMYB111*-F: | 5'-TTTCAGAGCGGATCCGAATTCATGGGAAGAGCGCCGCGA-3' |
| pET-28a-*WmMYB111*-R: | 5'-TGCGGCCGCAAGCTTGTCGACGTAGTTCAGATAAGGAAATAGTCTGTCATAA-3' |
| pGEX-*WmABF1-1*-F: | 5'-CCGCGTGGATCCCCGGAATTCATGGTTTCGCAGACAGAGTCAAG-3' |
| pGEX-*WmABF1-1*-R: | 5'-GATGCGGCCGCTCGAGTCGACCCAAGGACCTGTTAAGGTTCTTC-3' |
| Probe-*pWmNCED6*-MBS2-F: | 5'-GCCAAAAAGATCCTCATGGTAGGTATCCCGCGAATCCGCA-3' |
| Probe-*pWmNCED6*-MBS2-R: | 5'-TGCGGATTCGCGGGATACCTACCATGAGGATCTTTTTGGC-3' |
| Probe-*pWmNCED9*-MBS-F: | 5'-CAGGCAGTGGGCCTCCTTAGGTACACAACATTTCTGTTCC-3' |
| Probe-*pWmNCED9*-MBS-R: | 5'-GGAACAGAAATGTTGTGTACCTAAGGAGGCCCACTGCCTG-3' |
| Probe-*pWmSEN1*-MBS2-F: | 5'-TTACAATACTTAGCATTCTAGGTAATTTACATCCAGGAAT-3' |
| Probe-*pWmSEN1*-MBS2-R: | 5'-ATTCCTGGATGTAAATTACCTAGAATGCTAAGTATTGTAA-3' |
| Probe-*pWmSEN2*-MBS2-F: | 5'-TTGTAACTTTTAAGGTCTTAGGTTCAAGCACTTTAATGTG-3' |
| Probe-*pWmSEN2*-MBS2-R: | 5'-CACATTAAAGTGCTTGAACCTAAGACCTTAAAAGTTACAA-3' |
| Probe-*pWmPAO1-1*-MBS1-F: | 5'-CAAACTTTATACTAAAATTAGGTATTCCAAAATGAAATAA-3' |
| Probe-*pWmPAO1-1*-MBS1-R: | 5'-TTATTTCATTTTGGAATACCTAATTTTAGTATAAAGTTTG-3' |
| Probe-*pWmPAO2-1*-MBS4-F: | 5'-ATCGCCATTCAATCTTGTTAGGTCATGGTCTCGCTAAATC-3' |
| Probe-*pWmPAO2-1*-MBS4-R: | 5'-GATTTAGCGAGACCATGACCTAACAAGATTGAATGGCGAT-3' |
| Probe-*pWmNRT1.7a*-MBS1-F: | 5'-GTCCAGATGGTACATAGACCTAATTTCGTGAGTTAAAAGA-3' |
| Probe-*pWmNRT1.7a*-MBS1-R: | 5'-TCTTTTAACTCACGAAATTAGGTCTATGTACCATCTGGAC-3' |
| Probe-*pWmNRT1.7a*-ABS1-F: | 5'-ACAAAACATAGCAATAAACGTGGAAAAAATTTGAATGAAT-3' |
| Probe-*pWmNRT1.7a*-ABS1-R: | 5'-ATTCATTCAAATTTTTTCCACGTTTATTGCTATGTTTTGT-3' |
| Probe-*pWmNRT2.5*-MBS2-F: | 5'-CGCCCACAGGCTTCATTTTGGTGCGCTTCTTCATCGGATT-3' |
| Probe-*pWmNRT2.5*-MBS2-R: | 5'-AATCCGATGAAGAAGCGCACCAAAATGAAGCCTGTGGGCG-3' |
| Probe-*pWmNRT2.5*-ABS2-F: | 5'-GTAAAACACTTTAATTACACGTGTAAATCATTTAGTAAAA-3' |
| Probe-*pWmNRT2.5*-ABS2-R: | 5'-TTTTACTAAATGATTTACACGTGTAATTAAAGTGTTTTAC-3' |
| Probe-*pWmMYB111-*ABS3-F: | 5'-AAATTTACTTAATTAAGACGTGTCCTTGCATCAAAATCAT-3' |
| Probe-*pWmMYB111*-ABS3-R: | 5'-ATGATTTTGATGCAAGGACACGTCTTAATTAAGTAAATTT-3' |
|  |  |

| **Supplemental Table S6. Primers used for Y1H** | |  |
| --- | --- | --- |
| **Primer name** | **Sequence** |  |
| pGADT7*-WmMYB111*-F: | 5'-GCCATGGAGGCCAGTGAATTCATGGGAAGAGCGCCGCGA-3' | |
| pGADT7-*WmMYB111*-R: | 5'-ACGATTCATCTGCAGCTCGAGTTAGTAGTTCAGATAAGGAAATAGTCTGTCA-3' | |
| *pWmNCED6:AbAi*-a-F: | 5'-CTTGAATTCGAGCTCGGTACCGATTTGACACGTGCCTTTGCT-3' | |
| *pWmNCED6:AbAi*-a-R: | 5'-ATACAGAGCACATGCCTCGAGACCCGGTATTTGTTCATTTGAAG-3' | |
| *pWmNCED9:AbAi*-b-F: | 5'-CTTGAATTCGAGCTCGGTACCTGGATTACGGATATCGACTTCAAC-3' | |
| *pWmNCED9:AbAi*-b-R: | 5'-ATACAGAGCACATGCCTCGAGTTATTAAAACGTTCGTCTTACTATGACC-3' | |
| *pWmSEN1:AbAi*-c-F: | 5'-CTTGAATTCGAGCTCGGTACCTGAATGGACTTGAGTTTGTACAACC-3' | |
| *pWmSEN1:AbAi*-c-R: | 5'-ATACAGAGCACATGCCTCGAGAACTGAATAAGGATTGTTTGACCTATG-3' | |
| *pWmSEN2:AbAi*-d-F: | 5'-CTTGAATTCGAGCTCGGTACCTCTGCACAATTCAAATTCTACACATC-3' | |
| *pWmSEN2:AbAi*-d-R: | 5'-ATACAGAGCACATGCCTCGAGGAATGTCCGATCAAGTGAACTCTAAG-3' | |
| *pWmPAO1-1:AbAi*-e-F: | 5'-CTTGAATTCGAGCTCGGTACCAAAGAAATAAAAGTAATCCACTAAAGCTG-3' | |
| *pWmPAO1-1:AbAi*-e-R: | 5'-ATACAGAGCACATGCCTCGAGAAGGTATCACTTTGGCTACACATTATC-3' | |
| *pWmPAO2-1:AbAi*-f-F: | 5'-CTTGAATTCGAGCTCGGTACCTTTAGGTTATTTTTTGATTGGAAATTC-3' | |
| *pWmPAO2-1:AbAi*-f-R: | 5'-ATACAGAGCACATGCCTCGAGCACGACATCCTTGTGATAAATGG-3' | |
| pGADT7*-WmABF1-1*-F: | 5'-GCCATGGAGGCCAGTGAATTCTGGTTTCGCAGACAGAGTCAAG-3' | |
| pGADT7-*WmABF1-1*-R: | 5'-ACGATTCATCTGCAGCTCGAGTCACCAAGGACCTGTTAAGGTTC-3' | |
| *pWmMYB111:AbAi*-P1-F: | 5'-CTTGAATTCGAGCTCGGTACCCCATAATAACTAGTCTAGCAATATTGCATT-3' | |
| *pWmMYB111:AbAi*-P1-R: | 5'-ATACAGAGCACATGCCTCGAGCAATACATATCTCTATAGAACATAAAGTAACTCG-3' | |
| *pWmMYB111:AbAi*-P2-F: | 5'-CTTGAATTCGAGCTCGGTACCGTGAGTAAGTGCTATCCCATCTTATTAAC-3' | |
| *pWmMYB111:AbAi*-P2-R: | 5'-ATACAGAGCACATGCCTCGAGTTTCCGCAGAAAGTCTCTCATC-3' | |
| *pWmMYB111:AbAi*-P3-F: | 5'-CTTGAATTCGAGCTCGGTACCTAGCTAATAGAAGCACACTGAATAACC-3' | |
| *pWmMYB111:AbAi*-P3-R: | 5'-ATACAGAGCACATGCCTCGAGCAGCGTTTTCACGCGCAA-3' | |
| *pWmNRT1.7a:AbAi*-P1-F: | 5'-CTTGAATTCGAGCTCGGTACCTAATTGTTGAATATAAATTTTTAAATTGAGTAG-3' | |
| *pWmNRT1.7a:AbAi*-P1-R: | 5'-ATACAGAGCACATGCCTCGAGGGTTTGCCTTCTATGTTTTAATATATTTG-3' | |
| *pWmNRT2.5:AbAi*-P2-F: | 5'-CTTGAATTCGAGCTCGGTACCGCTATAAAACTTTAAGGAACTTGTTTGG-3' | |
| *pWmNRT2.5:AbAi*-P2-R: | 5'-ATACAGAGCACATGCCTCGAGCTAAATGATTTACACGTGTAATTAAAGTG-3' | |
| *pWmNRT2.5:AbAi*-P3-F: | 5'-CTTGAATTCGAGCTCGGTACCGACCGGATCCGTCCTCTCG-3' | |
| *pWmNRT2.5:AbAi*-P3-R: | 5'-ATACAGAGCACATGCCTCGAGGGAGCTCATCCAGAACTGACAAC-3' | |
|  |  |  |

| **Supplemental Table S7. Primers used for Dual Luciferase assays** | |
| --- | --- |
| **Primer name** | **Sequence** |
| 62SK-*WmMYB111*-F: | 5'-AGGACAGCCCAAGCTGAGCTCATGGGAAGAGCGCCGCGA-3' |
| 62SK-*WmMYB111*-R: | 5'-TCAGCGTACCGAATTGGTACCTTAGTAGTTCAGATAAGGAAATAGTCTGTCA-3' |
| *pWmNCED6:LUC*-a-F: | 5'-CTATAGGGCGAATTGGGTACCGATTTGACACGTGCCTTTGCT-3' |
| *pWmNCED6:LUC*-a-R: | 5'-CGCTCTAGAACTAGTGGATCCGGATCCACCCGGTATTTGTTCATTTGAAG-3' |
| *pWmNCED9:LUC*-b-F: | 5'-CTATAGGGCGAATTGGGTACCTGGATTACGGATATCGACTTCAAC-3' |
| *pWmNCED9:LUC*-b-R: | 5'-CGCTCTAGAACTAGTGGATCCGGATCCTTATTAAAACGTTCGTCTTACTATGACC-3' |
| *pWmSEN1:LUC*-c-F: | 5'-CTATAGGGCGAATTGGGTACCTGAATGGACTTGAGTTTGTACAACC-3' |
| *pWmSEN1:LUC*-c-R: | 5'-CGCTCTAGAACTAGTGGATCCGGATCCAACTGAATAAGGATTGTTTGACCTATG-3' |
| *pWmSEN2:LUC*-d-F: | 5'-CTATAGGGCGAATTGGGTACCTCTGCACAATTCAAATTCTACACATC-3' |
| *pWmSEN2:LUC*-d-R: | 5'-CGCTCTAGAACTAGTGGATCCGGATCCGAATGTCCGATCAAGTGAACTCTAAG-3' |
| *pWmPAO1-1:LUC*-e-F: | 5'-CTATAGGGCGAATTGGGTACCAAAGAAATAAAAGTAATCCACTAAAGCTG-3' |
| *pWmPAO1-1:LUC*-e-R: | 5'-CGCTCTAGAACTAGTGGATCCGGATCCAAGGTATCACTTTGGCTACACATTATC-3' |
| *pWmPAO2-1:LUC*-f-F: | 5'-CTATAGGGCGAATTGGGTACCTTTAGGTTATTTTTTGATTGGAAATTC-3' |
| *pWmPAO2-1:LUC*-f-R: | 5'-CGCTCTAGAACTAGTGGATCCGGATCCCACGACATCCTTGTGATAAATGG-3' |
| 62SK-*WmABF1-1*-F: | 5'-AGGACAGCCCAAGCTGAGCTCTGGTTTCGCAGACAGAGTCAAG-3' |
| 62SK-*WmABF1-1*-R: | 5'-TCAGCGTACCGAATTGGTACCTCACCAAGGACCTGTTAAGGTTC-3' |
| *pWmMYB111:LUC*-P1-F: | 5'-CTATAGGGCGAATTGGGTACCCCATAATAACTAGTCTAGCAATATTGCATT-3' |
| *pWmMYB111:LUC*-P1-R: | 5'-CGCTCTAGAACTAGTGGATCCCAATACATATCTCTATAGAACATAAAGTAACTCG-3' |
| *pWmMYB111:LUC*-P2-F: | 5'-CTATAGGGCGAATTGGGTACCGTGAGTAAGTGCTATCCCATCTTATTAAC-3' |
| *pWmMYB111:LUC*-P2-R: | 5'-CGCTCTAGAACTAGTGGATCCTTTCCGCAGAAAGTCTCTCATC-3' |
| *pWmMYB111:LUC*-P3-F: | 5'-CTATAGGGCGAATTGGGTACCTAGCTAATAGAAGCACACTGAATAACCTA-3' |
| *pWmMYB111:LUC*-P3-R: | 5'-CGCTCTAGAACTAGTGGATCCCAGCGTTTTCACGCGCAA-3' |
| *pWmNCED6:LUC*-A-F: | 5'-CTATAGGGCGAATTGGGTACCCCAACAACATATGACATATGTAAATCAA-3' |
| *pWmNCED6:LUC*-A-R: | 5'-CGCTCTAGAACTAGTGGATCCTGGCATCGCTGCCGCTTT-3' |
| *pWmSEN1:LUC*-B-F: | 5'-CTATAGGGCGAATTGGGTACCACTACATTTAGCATTTTCATTACAATACCT-3' |
| *pWmSEN1:LUC*-B-R: | 5'-CGCTCTAGAACTAGTGGATCCACATTATTTAAGGGAAGAAAATTATCCTC-3' |
| *pWmPAO1-1:LUC*-C-F: | 5'-CTATAGGGCGAATTGGGTACCAAAGTGATACCTTTAATCGTTCACGT-3' |
| *pWmPAO1-1:LUC*-C-R: | 5'-CGCTCTAGAACTAGTGGATCCTCAAGAACTTCGGTTGGTGGTC-3' |
| *pWmNRT1.7a:LUC*-P1-F: | 5'-CTATAGGGCGAATTGGGTACCTAATTGTTGAATATAAATTTTTAAATTGAGTAG-3' |
| *pWmNRT1.7a:LUC*-P1-R: | 5'-CGCTCTAGAACTAGTGGATCCGGTTTGCCTTCTATGTTTTAATATATTTG-3' |
| *pWmNRT2.5:LUC*-P2-F: | 5'-CTATAGGGCGAATTGGGTACCGCTATAAAACTTTAAGGAACTTGTTTGG-3' |
| *pWmNRT2.5:LUC*-P2-R: | 5'-CGCTCTAGAACTAGTGGATCCCTAAATGATTTACACGTGTAATTAAAGTG-3' |
| *pWmNRT2.5:LUC*-P3-F: | 5'-CTATAGGGCGAATTGGGTACCGACCGGATCCGTCCTCTCG-3' |
| *pWmNRT2.5:LUC*-P3-R: | 5'-CGCTCTAGAACTAGTGGATCCGGAGCTCATCCAGAACTGACAAC-3' |
|  |  |

| **Supplemental Table S8. Primers used for Y2H and BiFC** | |
| --- | --- |
| **Primer name** | **Sequence** |
| pGADT7-*WmABF1-1*-F: | 5'-GCCATGGAGGCCAGTGAATTCATGGTTTCGCAGACAGAGTCAAG-3' |
| pGADT7-*WmABF1-1*-R: | 5'-ACGATTCATCTGCAGCTCGAGTCACCAAGGACCTGTTAAGGTTC-3' |
| pGBKT7-*WmABF1-1*-F: | 5'-ATGGCCATGGAGGCCGAATTCATGGTTTCGCAGACAGAGTCAAG-3' |
| pGBKT7-*WmABF1-1*-R: | 5'-CCGCTGCAGGTCGACGGATCCTCACCAAGGACCTGTTAAGGTTC-3' |
| pGADT7-*WmMYB111*-F: | 5'-GCCATGGAGGCCAGTGAATTCATGGGAAGAGCGCCGC-3' |
| pGADT7-*WmMYB111*-R: | 5'-ACGATTCATCTGCAGCTCGAGTTAGTAGTTCAGATAAGGAAATAGTCTGTCA-3' |
| pGBKT7-*WmMYB111*-F: | 5'-ATGGCCATGGAGGCCGAATTCATGGGAAGAGCGCCGC-3' |
| pGBKT7-*WmMYB111*-R: | 5'-CCGCTGCAGGTCGACGGATCCTTAGTAGTTCAGATAAGGAAATAGTCTGTCA-3' |
| YNE/YCE-*WmABF1-1*-F: | 5'-GATACATATGGGATCTACTAGTATGGTTTCGCAGACAGAGTCAAG-3' |
| YNE/YCE-*WmABF1-1*-R: | 5'-CATCCCGGGGGTACCGTCGACCCAAGGACCTGTTAAGGTTCTTC-3' |
| YNE/YCE-*WmMYB111*-F: | 5'-GATACATATGGGATCTACTAGTATGGGAAGAGCGCCGCGA-3' |
| YNE/YCE-*WmMYB111*-R: | 5'-CATCCCGGGGGTACCGTCGACGTAGTTCAGATAAGGAAATAGTCTGTCATAA-3' |
|  |  |
